# Supplementary material for: Design and Synthesis of Novel N-Benzylidene Derivatives of 3-Amino-4-imino-3,5-dihydro-4H-chromeno[2,3-d]pyrimidine under Microwave, In Silico ADME Predictions, In Vitro Antitumoral Activities and In Vivo Toxicity
Source: Pharmaceuticals (Basel). 2024 Apr 2;17(4):458. doi: 10.3390/ph17040458 (PMC11054303; doi:10.3390/ph17040458)

pharmaceuticals

Supporting Information

Design and synthesis of novel *N*-benzylidene derivatives of 3-amino-4-imino-3,5-dihydro-4*H*-chromeno[2,3-*d*]pyrimidine under microwave, *in silico* ADME predictions, *in vitro* antitumoral activities and *in vivo* toxicity.

Sirine Karoui [1,2], Marwa Dhiabi [1,2], Mehdi Fakhfakh [1], Souhir Abid [3], Emmanuelle Limanton [4], Rémy Le Guevel [5], Thierry D. Charlier [5,6], Anthony Mainguy [7], Olivier Mignen [7], Ludovic Paquin [2,4], Houcine Ammar [1] and Jean-Pierre Bazureau [2,4,*]

[1] *Laboratoire de Chimie Appliquée : Hétérocycles, Corps Gras et Polymères, Faculté des Sciences de Sfax, Université de Sfax, Route Soukra, BP 1171, 3000 Sfax, Tunisie.*
[2] *Institut des Sciences Chimiques de Rennes, ISCR UMR CNRS 6626, Université de Rennes 1, Campus de Beaulieu, Bât.10A, 263 Avenue du Général Leclerc, CS 74205, 35042 Rennes Cedex, France.*
[3] *Al-Jouf University, Department of Chemistry (Science and Arts): Al Qurayat, Al Jawf, Saudi Arabia.*
[4] *S2Wave platform, ScanMAT UAR 2025 CNRS, Université de Rennes 1, Campus de Beaulieu, Bât.10A, 263 Avenue du Général Leclerc, CS 74205, 35042 Rennes Cedex, France.*
[5] *ImPACcell platform, Biosit, SFR UMS CNRS 3480, Inserm 018, Campus de Villejean, Bât. 8, 2 Avenue du Prof. Léon Bernard, 35043 Rennes, France.*
[6] *Institut de Recherche en Santé, Environnement et Travail, IRSET Inserm UMR_S 1085, 9 Avenue du Professeur Léon Bernard, 35000 Rennes, France.*
[7] *Lymphocytes B & Auto-Immunité LBAI Inserm U1227, Université de Bretagne Occidentale, 22 Avenue Camille Desmoulins, 29200 Brest, France.*

* Correspondance : jean-pierre.bazureau@univ-rennes1.fr; Tel.: +33 223 236 603

Pages

Table S1: Bioavailability radar related to the physicochemical properties of compounds **10(a-l).** 2

Figure S1: BOILED-Egg model for compounds **10b**, **10e**, **10h** and **10k** (bearing a hydroxyl group) to predict gastrointestinal absorption (HIA-human gastrointestinal absorption) and BBB penetration

Figure S2: Images [a] of toxicity assays with erlotinib on Zebrafish *danio rerio* eleutheroembryos 3

Section 1: ^{1}H, ^{13}C NMR and MS spectra of compounds **10(a-l)** 4-27

Table S1: Bioavailability radar related to the physicochemical properties of compounds **10(a-l)** issued from SwissADME server [1-2]**.** The pink colored zone is the suitable physicochemical space for oral bioavailability. LIPO (Lipophilicity): -0.7 < Log P < +5.0; SIZE: 150 g/mol < MW < 500 g/mol; POLAR (Polarity): 20 Å^2 < *t*PSA < 130 Å^2; INSOLU (Insolubility): -6 < Log S < 0; INSATU (Insaturation): 0.25 < Fraction Csp^3 < 1; FLEX (Flexibility): 0 < Number rotatable bonds < 9.

Compound	Bioavailability radar [a]	Compound	Bioavailability radar [a]
**10a** 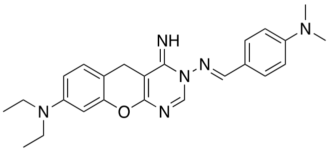	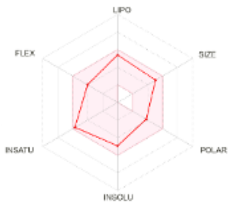	**10g** 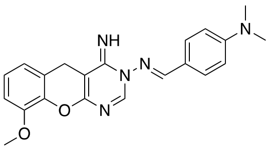	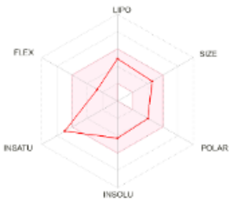
**10b** 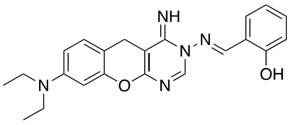	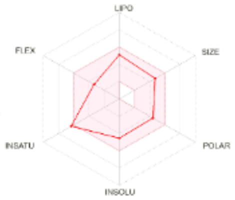	**10h** 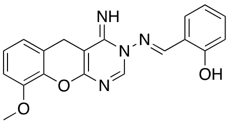	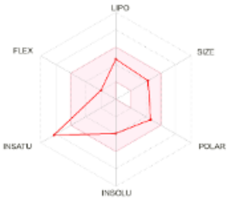
**10c** 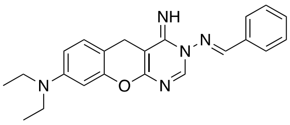	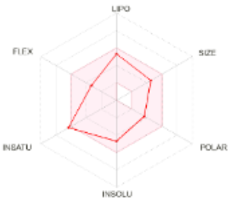	**10i** 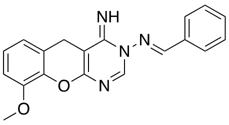	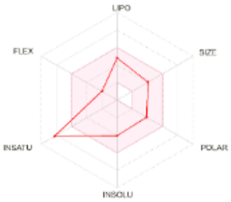
**10d** 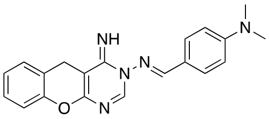	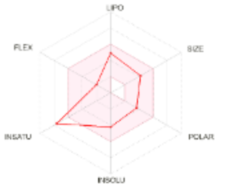	**10j** 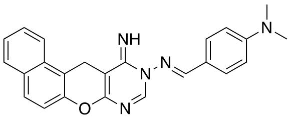	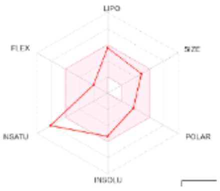
**10e** 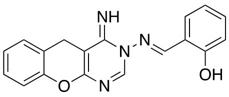	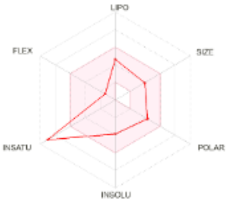	**10k** 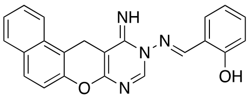	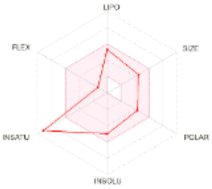
**10f** 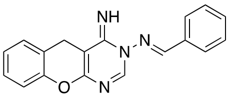	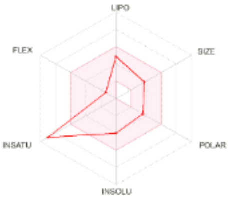	**10l** 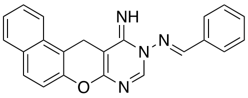	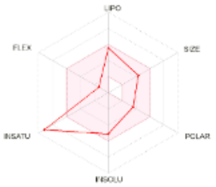

[a] %Abs: 55 for all compounds **10(a-l).**

Figure S1: BOILED-Egg model of compounds **10b**, **10e**, **10h** and **10k** (bearing a hydroxyl group, for comparison [a]) to predict gastrointestinal absorption (HIA-human gastrointestinal absorption) and BBB penetration in function of the position of the molecules in the *W*-Log P-*versus*-tPSA referential. The "white region" is for high probability of passive absorption by the gastrointestinal tract, and the "yellow region" (yolk) is for high probability of brain penetration. Yolk and white areas are not mutually exclusive. The "gray region" concerned compounds which are predicted not absorbed in gastrointestinal tract and not BBB permeant. PGP^+ in blue color means substrate of P-glycoprotein; PGP^- in red color means non-substrate of P-glycoprotein), issued from SwissADME server [1-2].

Compound		BOILED-Egg model (*W*-Log P-*versus*-tPSA)	Compound	
**10b**	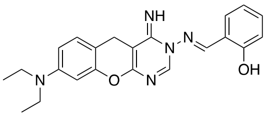	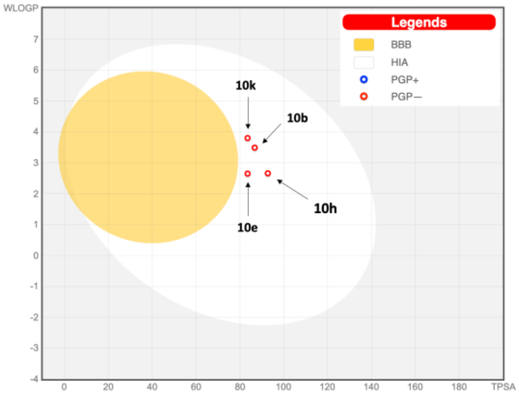	**10h**	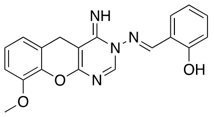
**10e**	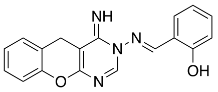		**10k**	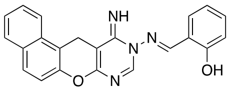

[a] Compounds **10b**, **10e**, **10h** and **10k** are not predicted to passive BBB penetration and are not effluxed by P-glycoprotein (PGP^-, red color).

Figure S2: Images [a] of toxicity assays with erlotinib on Zebrafish *danio rerio* eleutheroembryos (according to Fish Embryo Acute Toxicity (FET) Test, OCDE (2013) [3]).

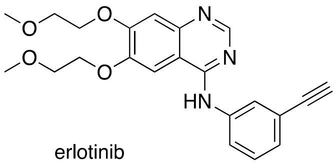

erlotinib

Dose of **erlotinib** (μM)	0 hpf	24 hpf	48 hpf	72 hpf	Dose of **erlotinib** (μM)	0 hpf	24 hpf	48 hpf	72 hpf
3	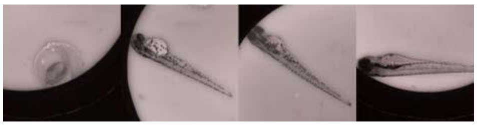				0.03	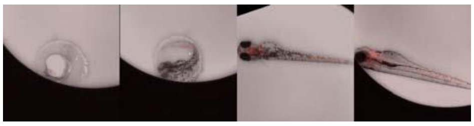			
1	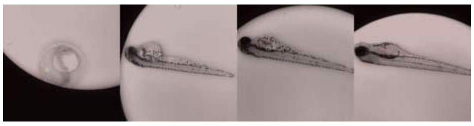				0.01	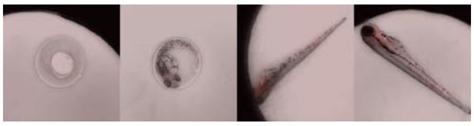			
0.33	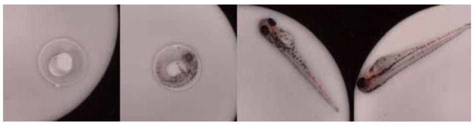				No treatment	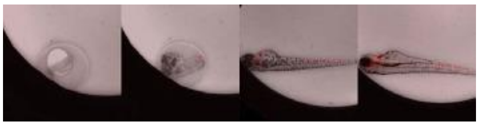			
0.11	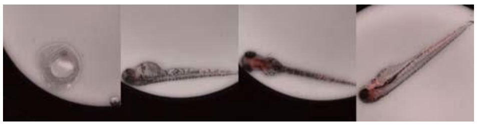								

[a] magnification: 2.5x

References:

1. Daina, A.; Michielin, O.; Zoete, V. SwissADME: a free web tool to. evaluate pharmacokinetics, druglikeness and medicinal chemistry friendliness of small molecules. *Nature Sc. Rep.* **2017**, *7*, 42717. doi: 10.1038/srep42717
2. Swiss ADME of Swiss Institute of Bioinformatics. Accessed 20 November 2023. http://www.swissadme.ch/
3. OECD. Test No. 236: Fish Embryo Acute Toxicity (FET) Test. OECD Guidelines for the testing of chemicals, Section 2. OECD Publishing: Paris, France, 2013.

^{1}H NMR (300 MHz, DMSO-d_6) spectrum of compound (**10a**).

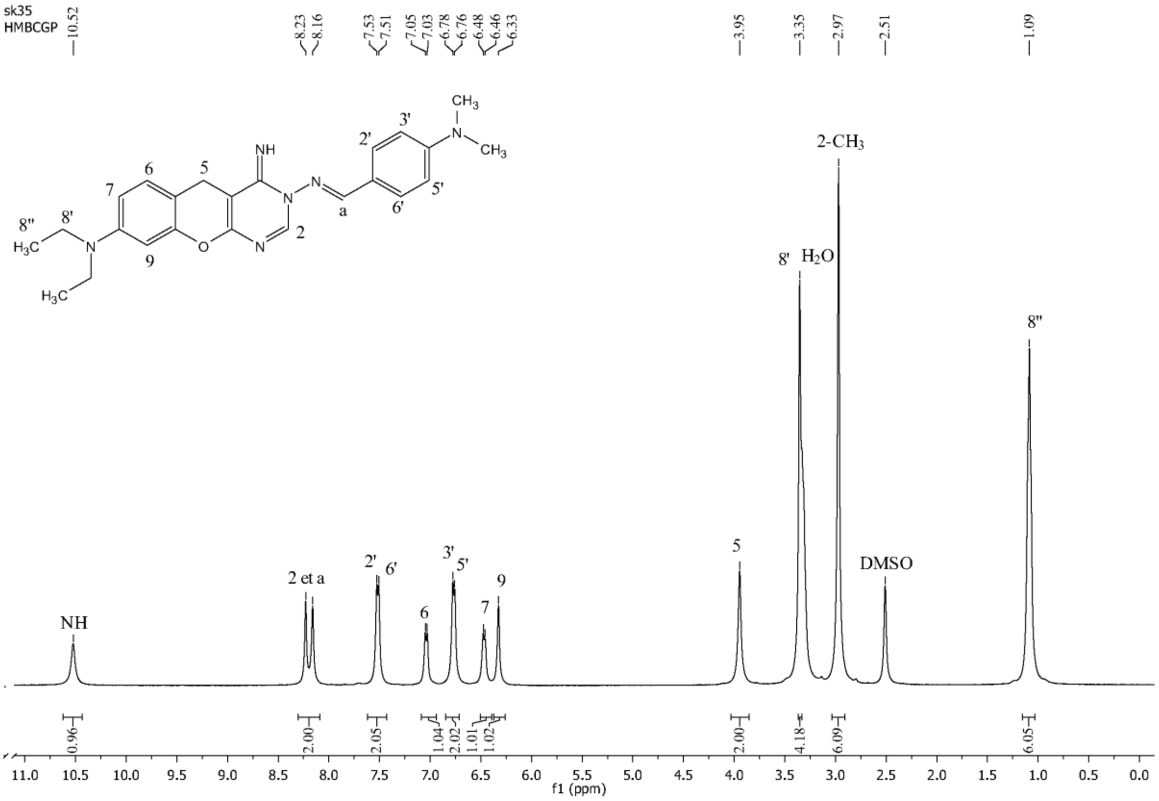

Details on the ^{1}H NMR (300 MHz, DMSO-d_6) spectrum of compound (**10a**).

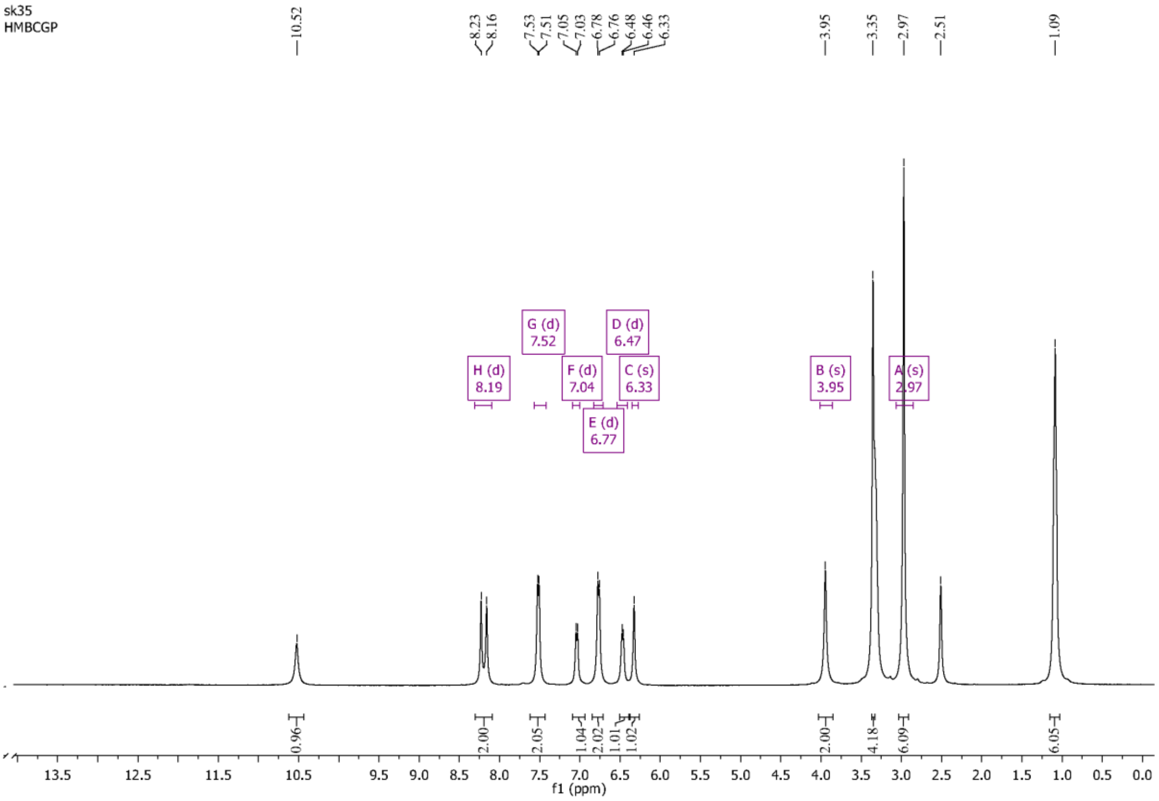

^{13}C NMR (75 MHz, DMSO-d_6) spectrum of compound (**10a**).

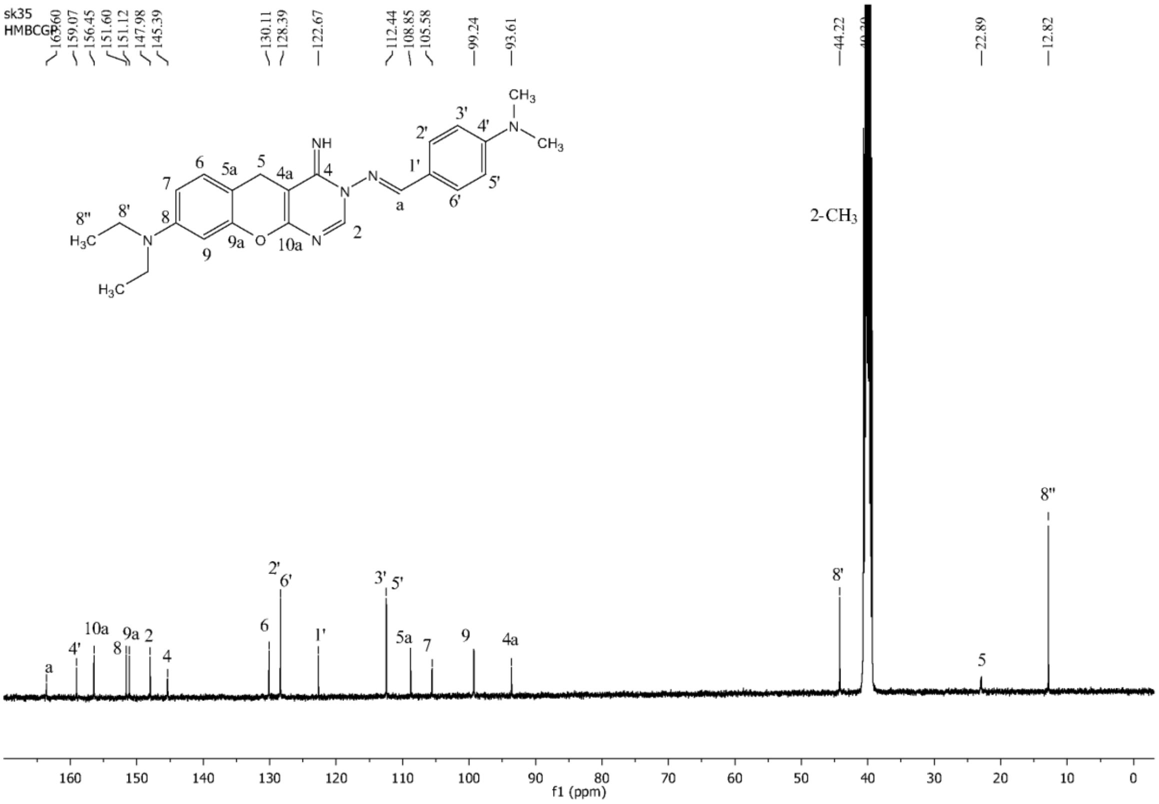

MS spectrum of compound (**10a**).

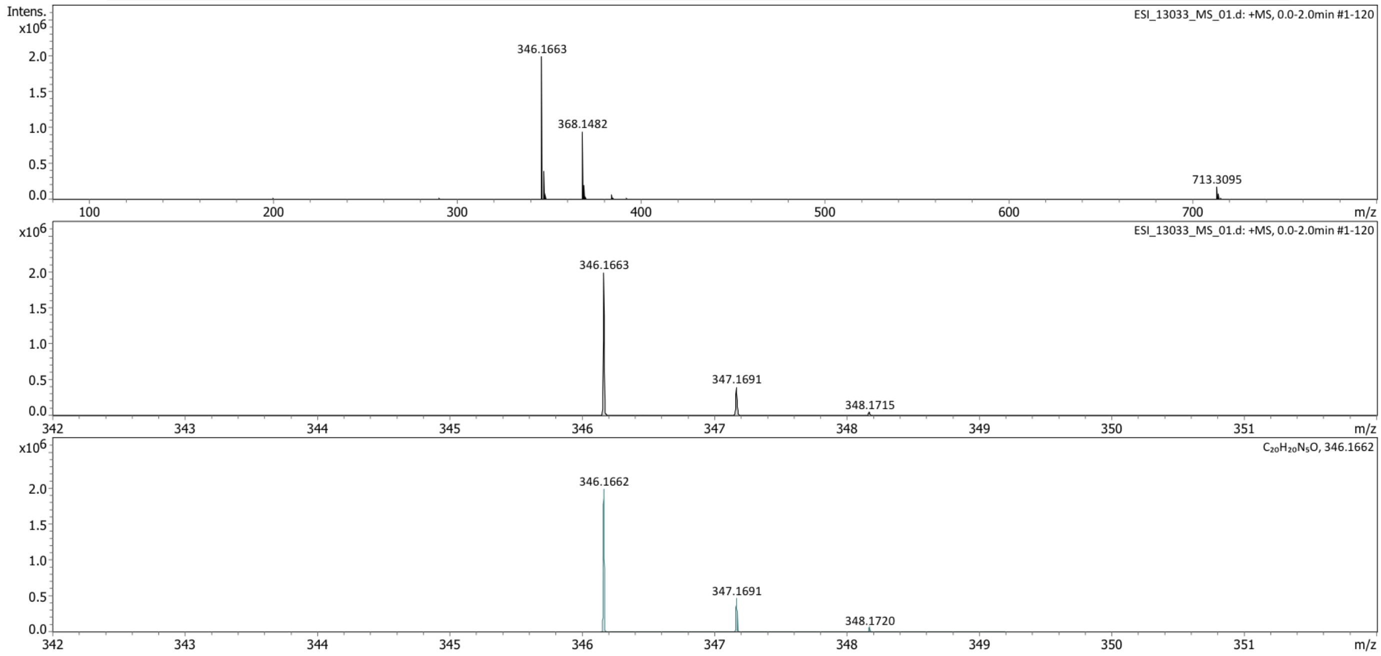

^{1}H NMR (300 MHz, DMSO-d_6) spectrum of compound (**10b**).

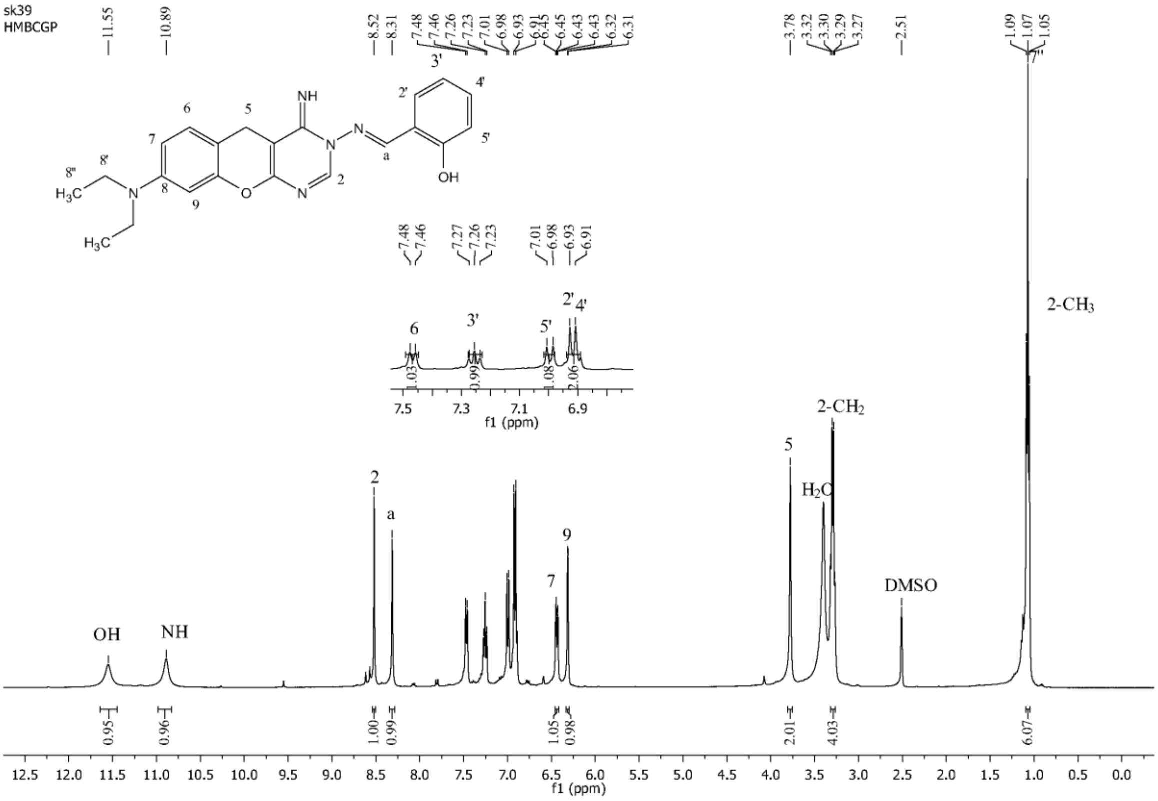

Details on the ^{1}H NMR (300 MHz, DMSO-d_6) spectrum of compound (**10b**).

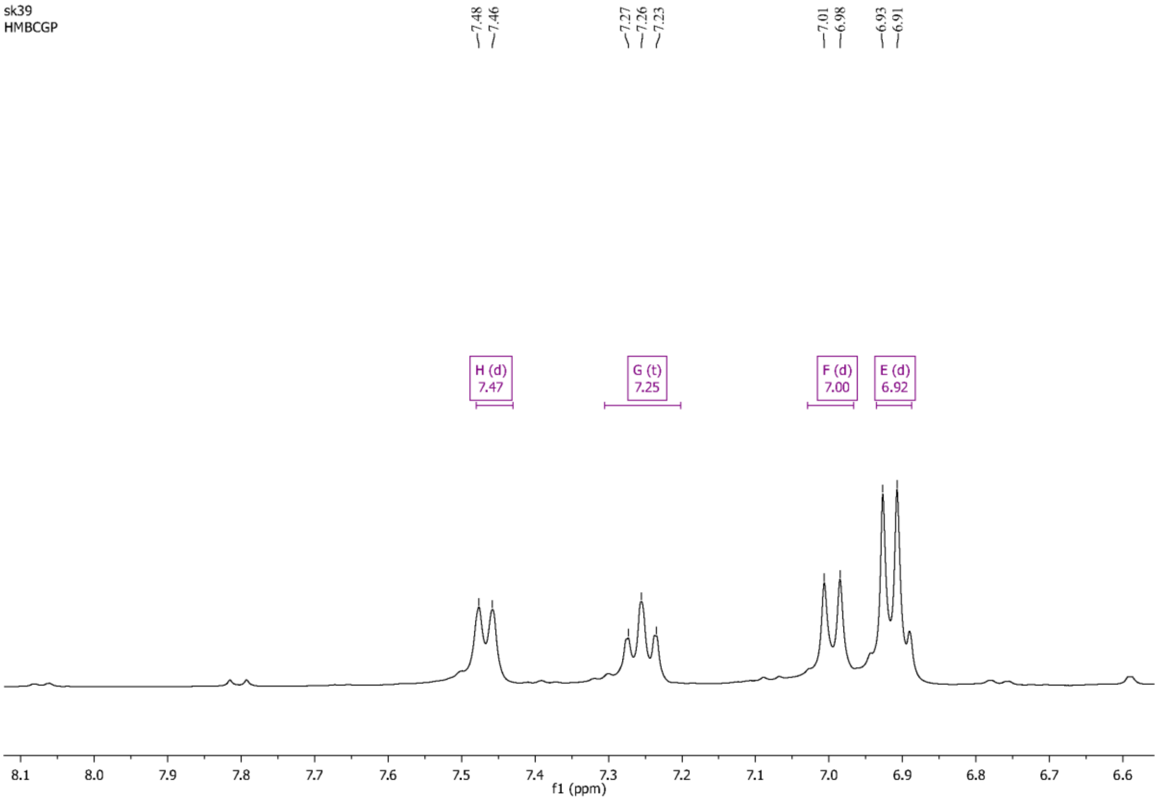

^{13}C NMR (75 MHz, DMSO-d_6) spectrum of compound (**10b**).

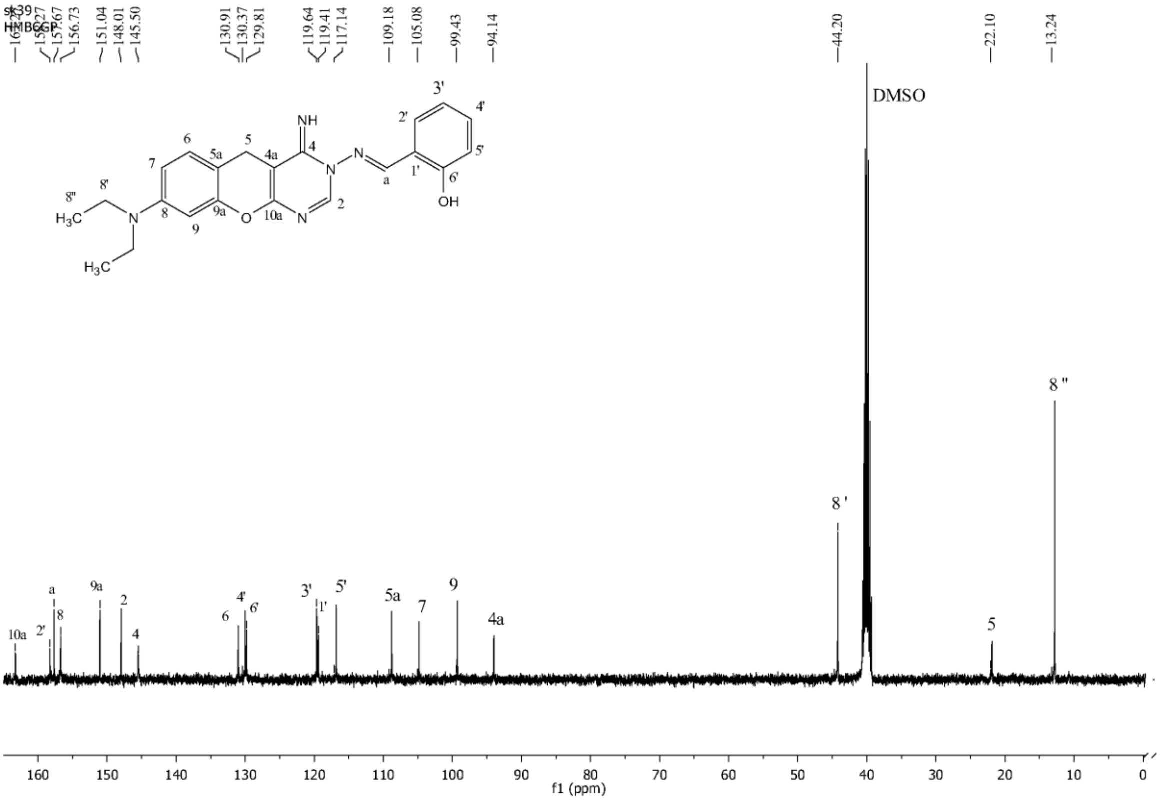

MS spectrum of compound (**10b**).

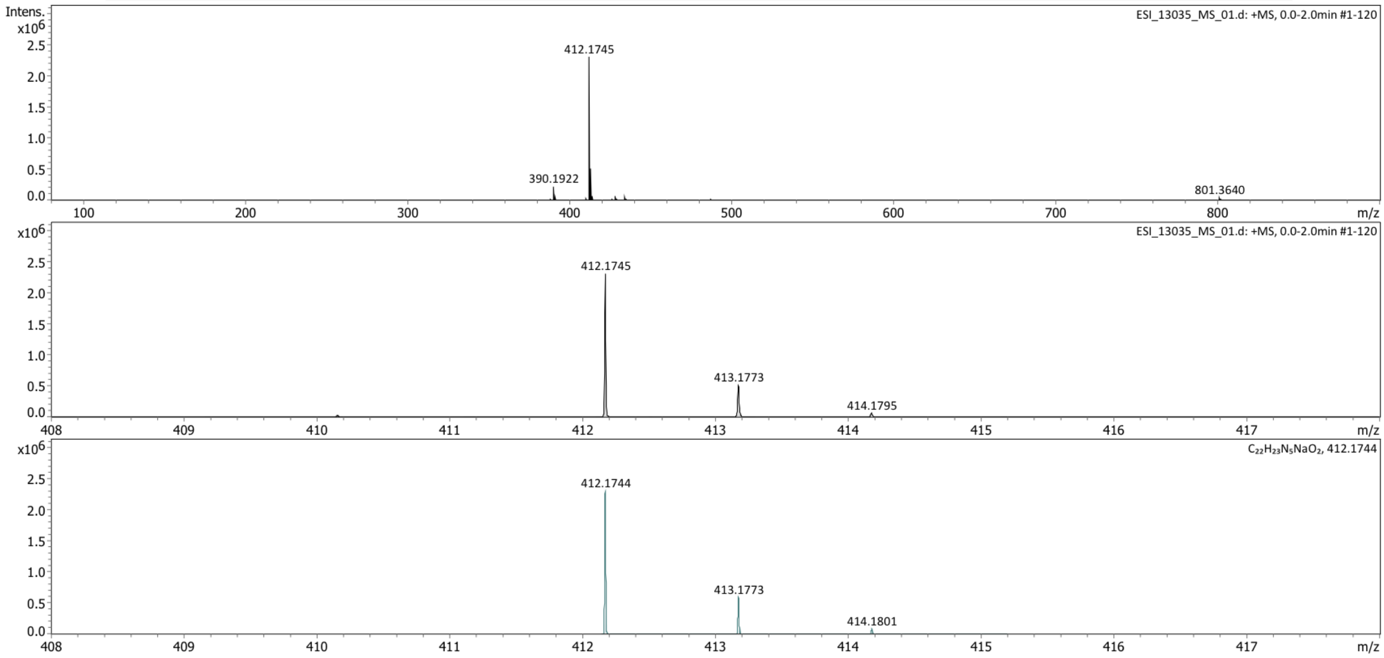

^{1}H NMR (300 MHz, DMSO-*d*$_6$) spectrum of compound (**10c**).

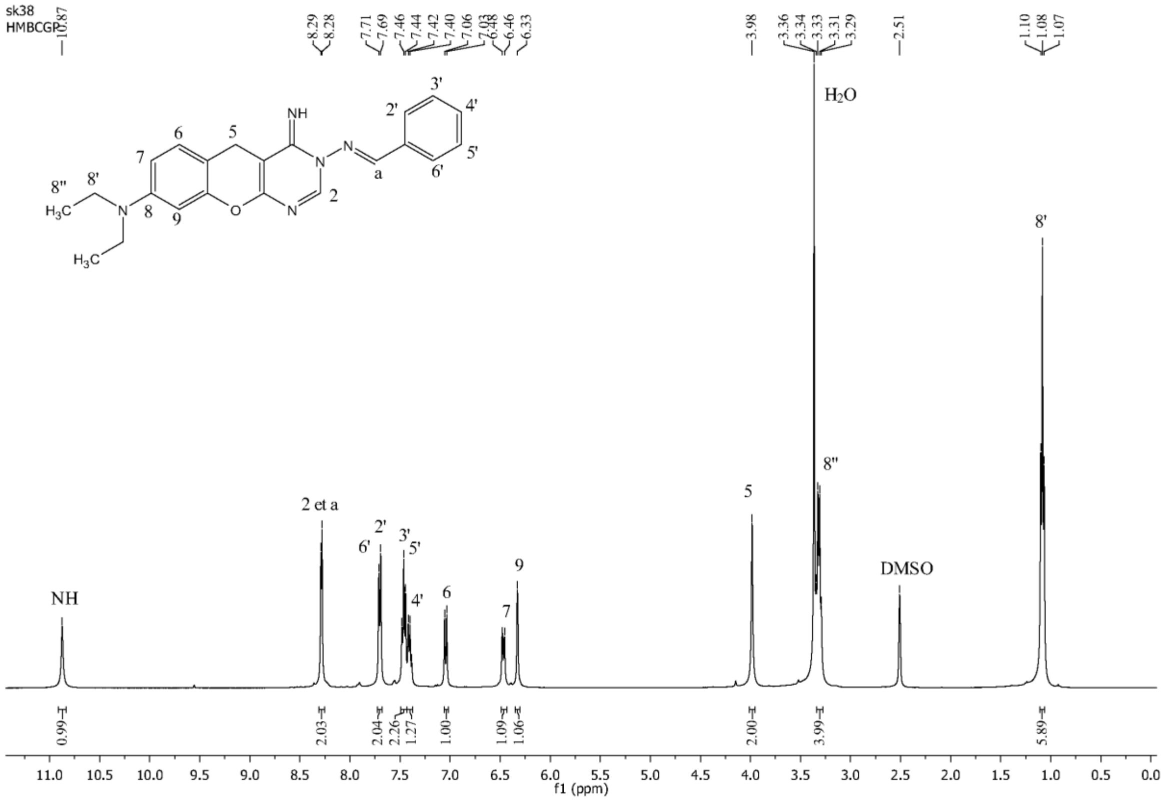

Details on the ^{1}H NMR (300 MHz, DMSO-*d*$_6$) spectrum of compound (**10c**).

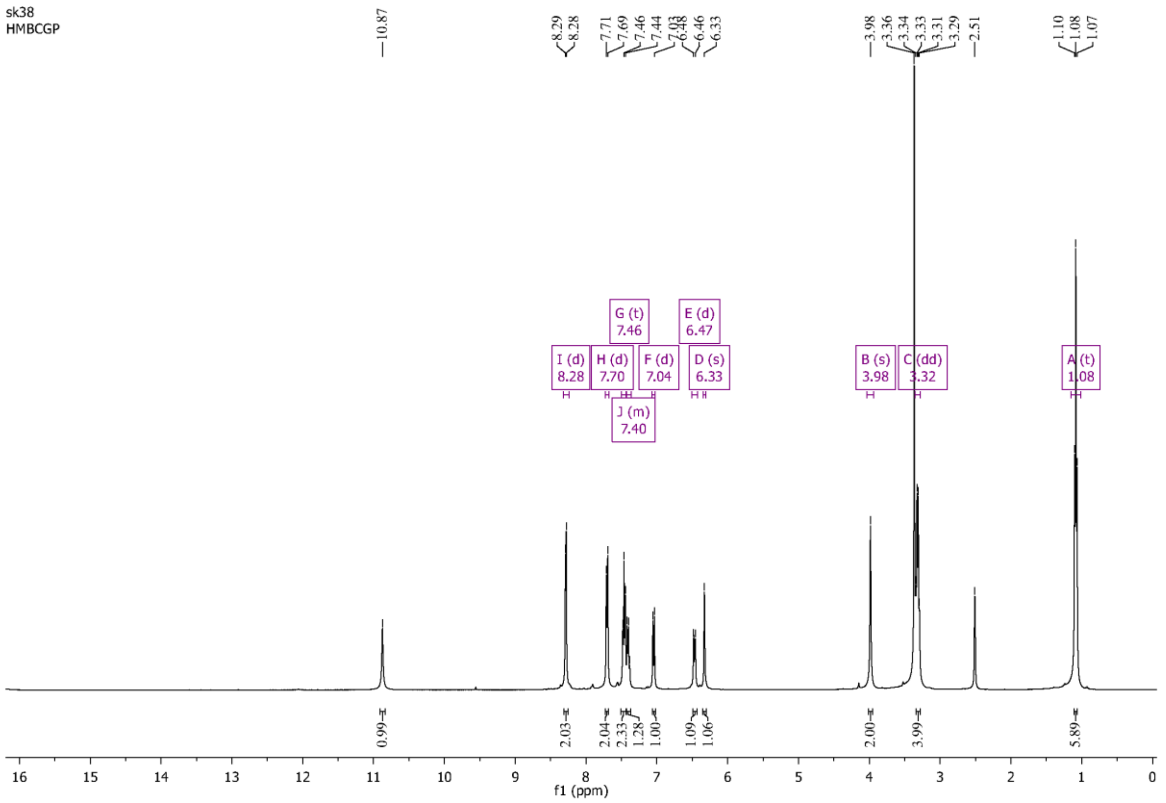

MS spectrum of compound (**10c**).

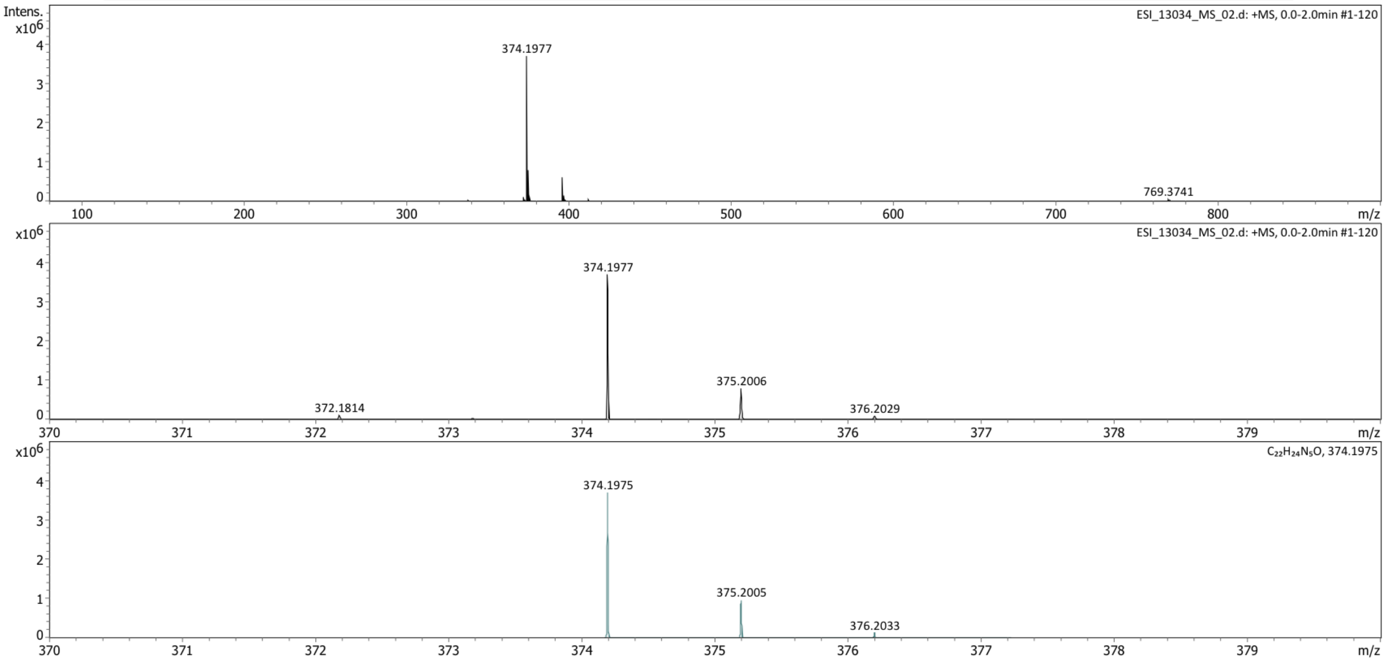

^{1}H NMR (300 MHz, DMSO-d_6) spectrum of compound (**10d**).

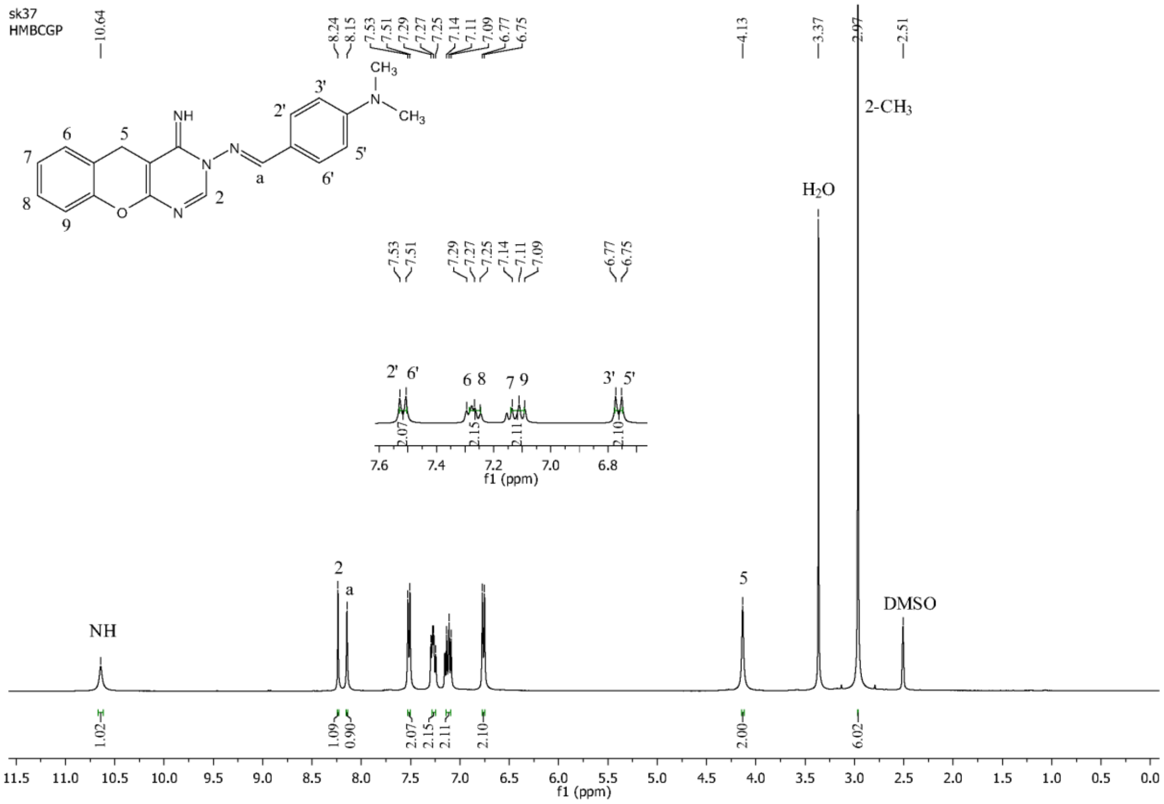

Details on the ^{1}H NMR (300 MHz, DMSO-d_6) spectrum of compound (**10d**).

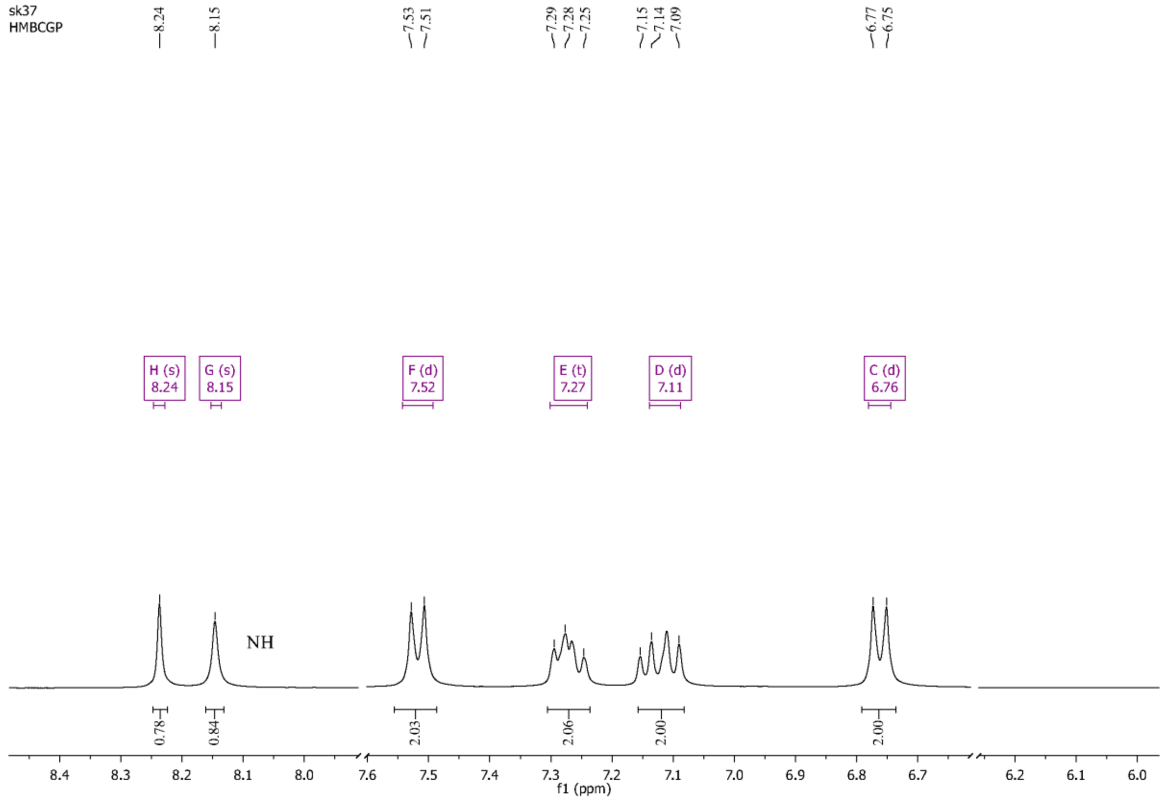

^{13}C NMR (75 MHz, DMSO-d_6) spectrum of compound (**10d**).

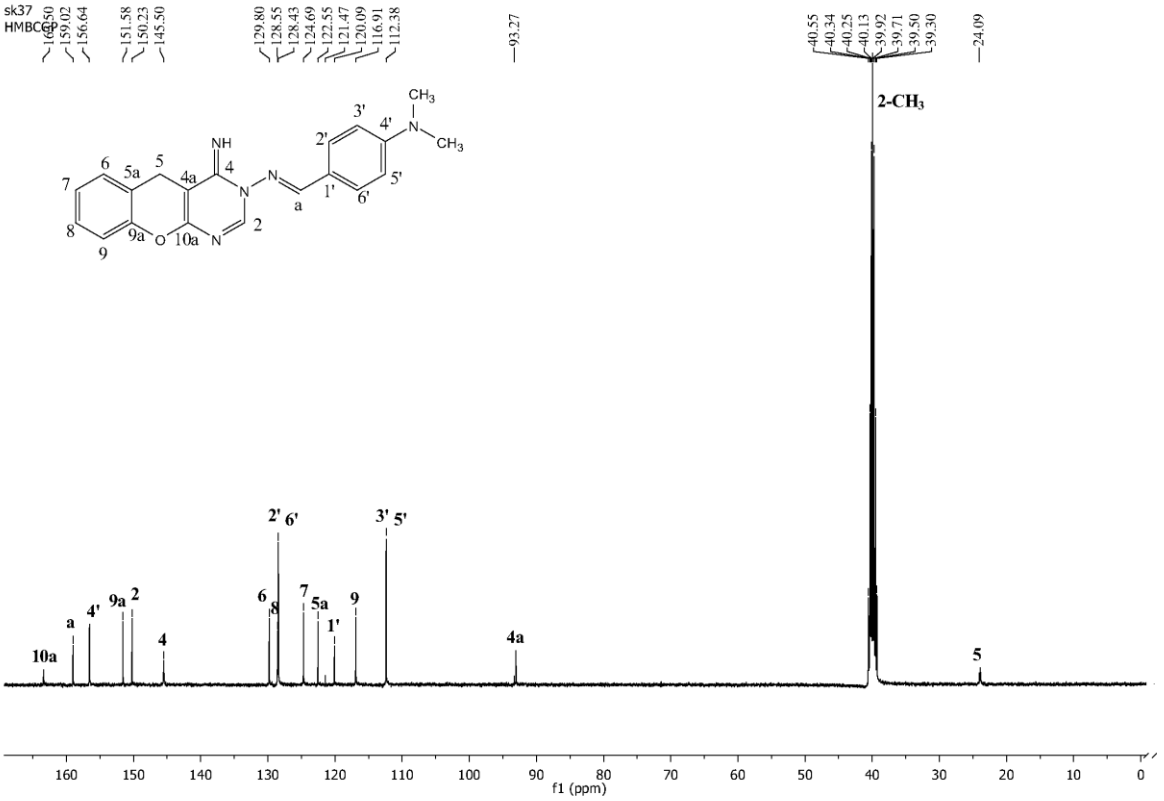

MS spectrum of compound (**10d**).

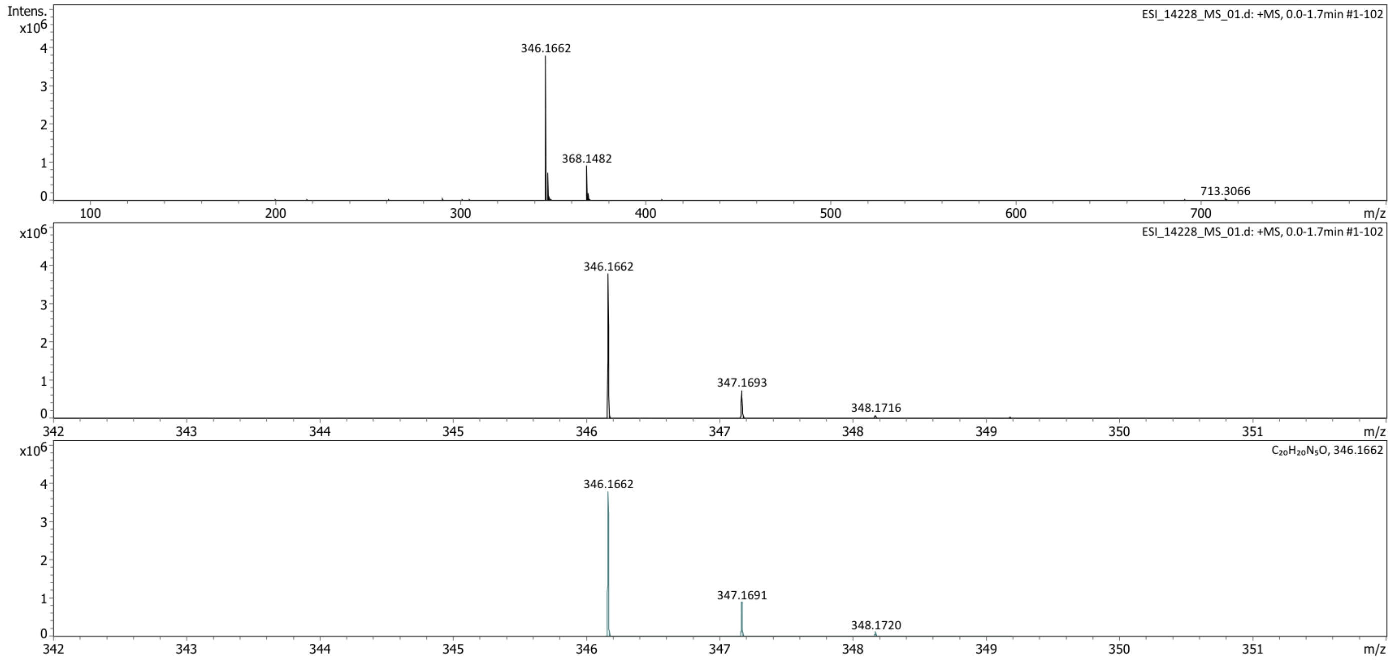

^{1}H NMR (300 MHz, DMSO-d_6) spectrum of compound (**10e**).

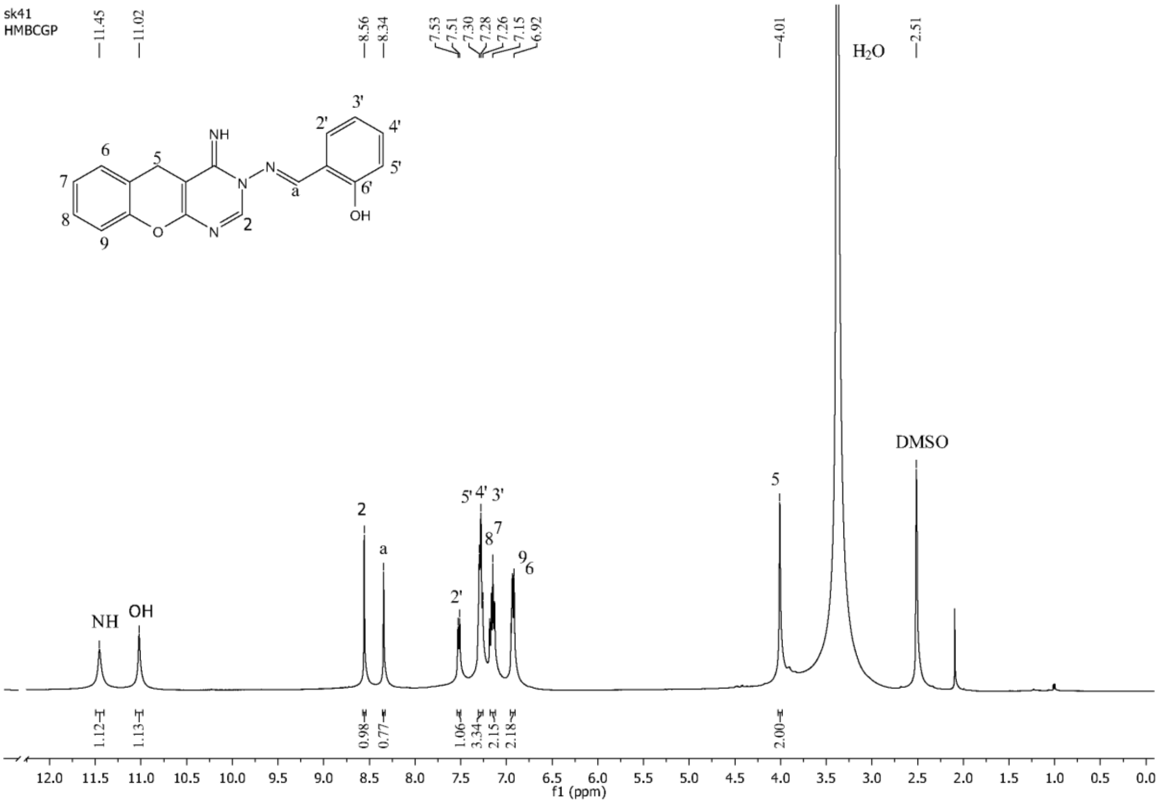

Details on the ^{1}H NMR (300 MHz, DMSO-d_6) spectrum of compound (**10e**).

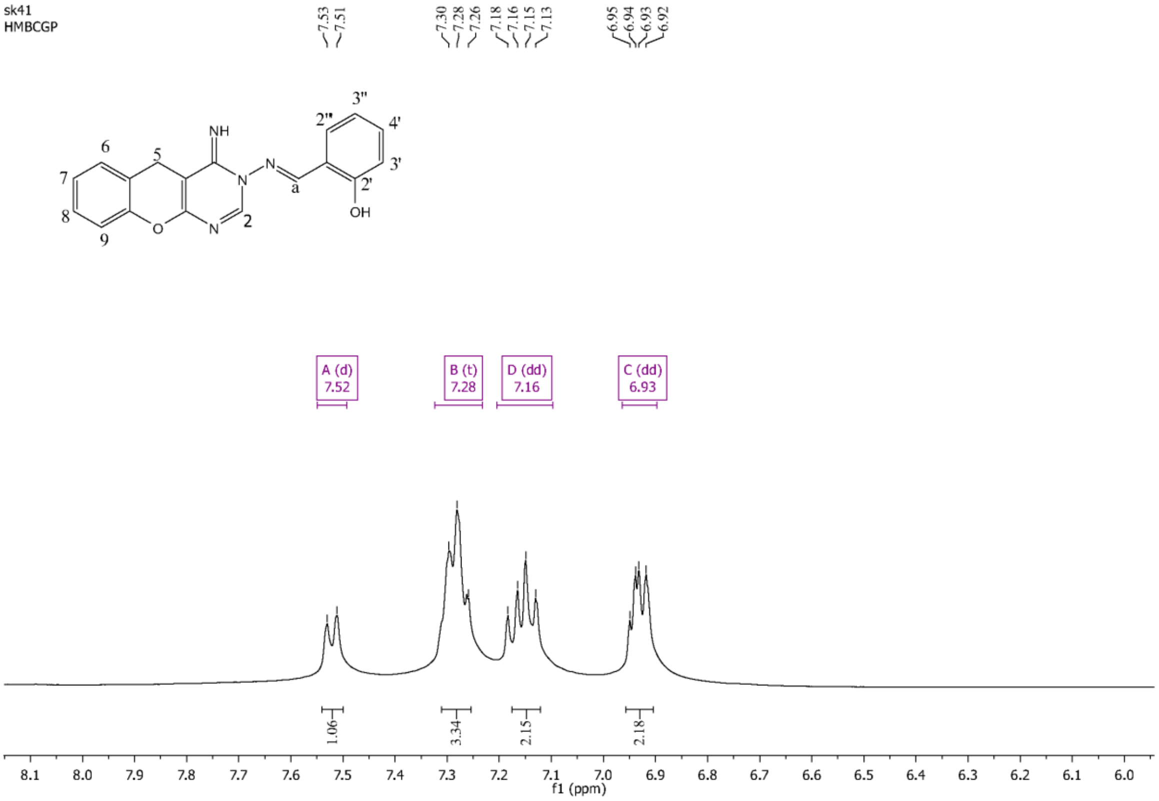

^{13}C NMR (75 MHz, DMSO-d_6) spectrum of compound (**10e**).

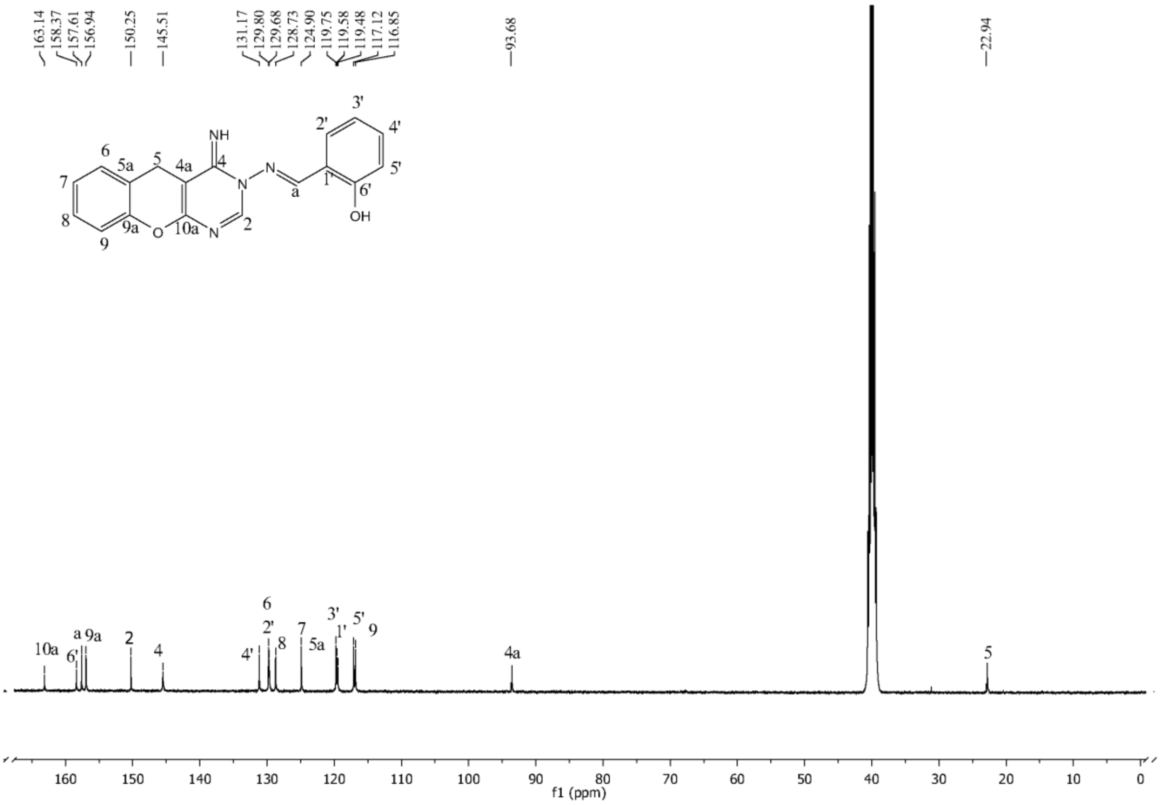

MS spectrum of compound (**10e**).

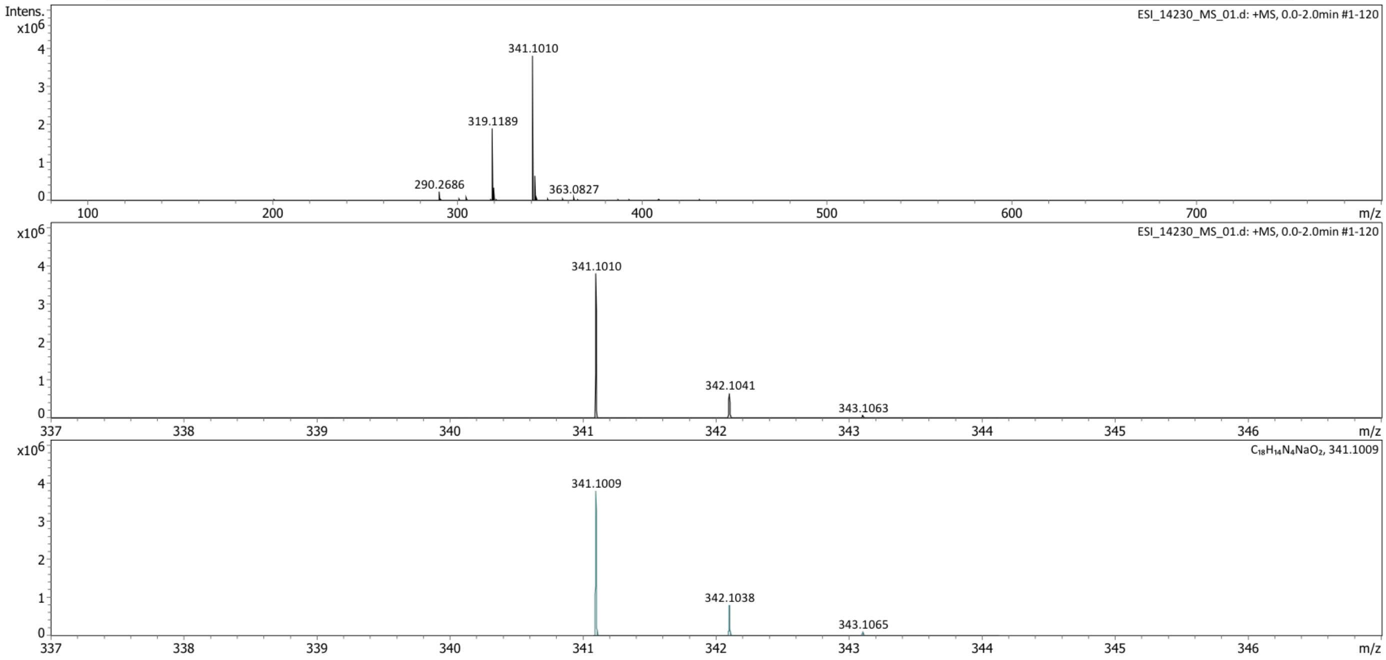

^{1}H NMR (300 MHz, DMSO-d_6) spectrum of compound (**10f**).

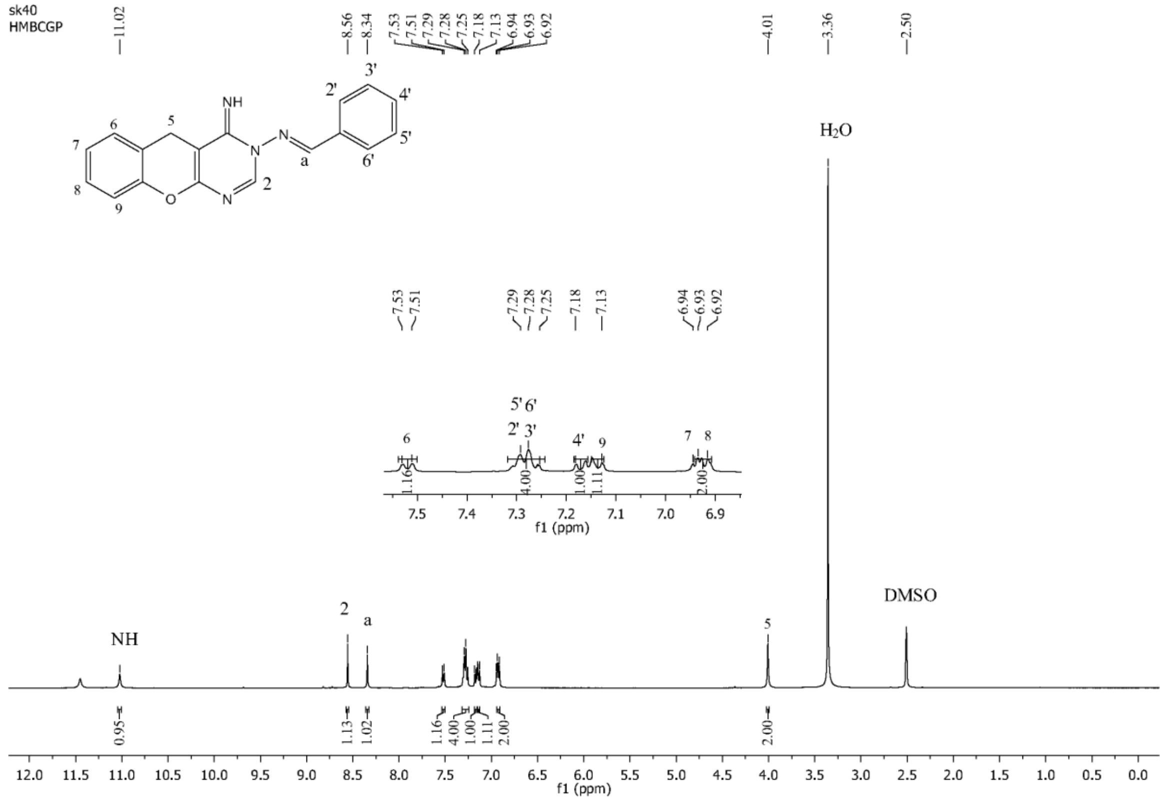

^{13}C NMR (75 MHz, DMSO-d_6) spectrum of compound (**10f**).

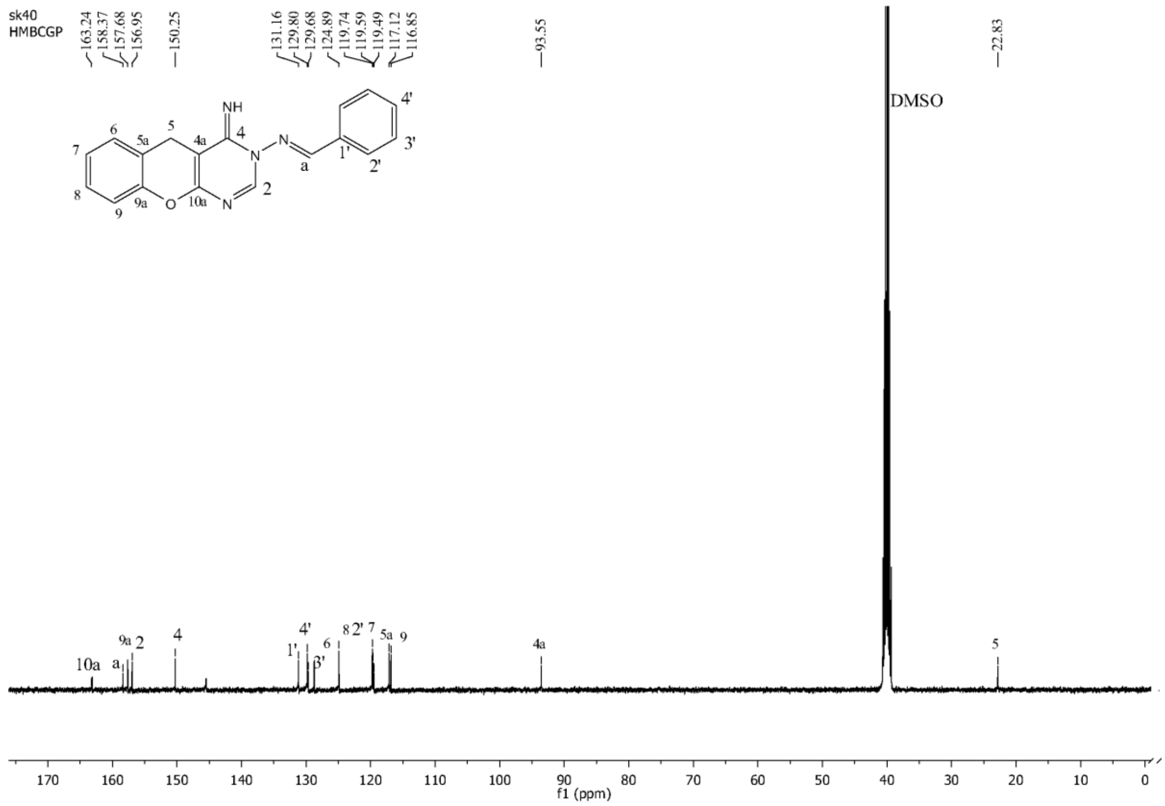

MS spectrum of compound (**10f**).

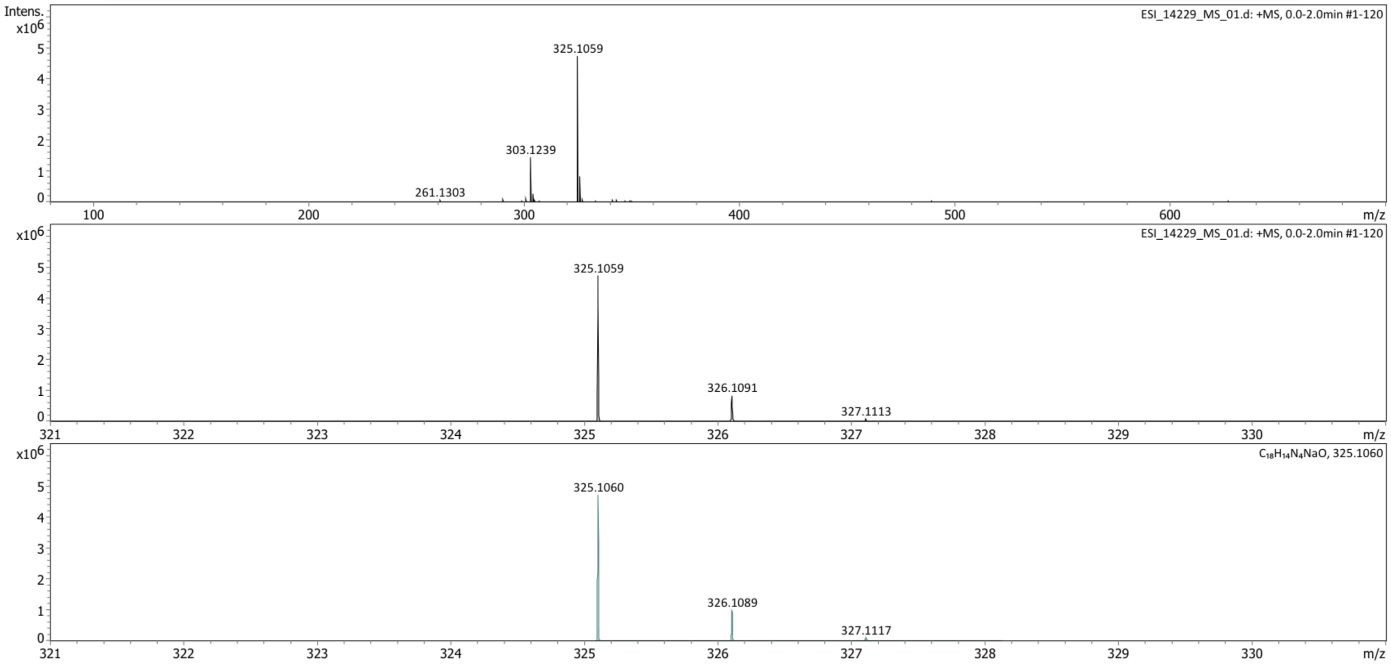

^{1}H NMR (300 MHz, DMSO-d_6) spectrum of compound (**10g**).

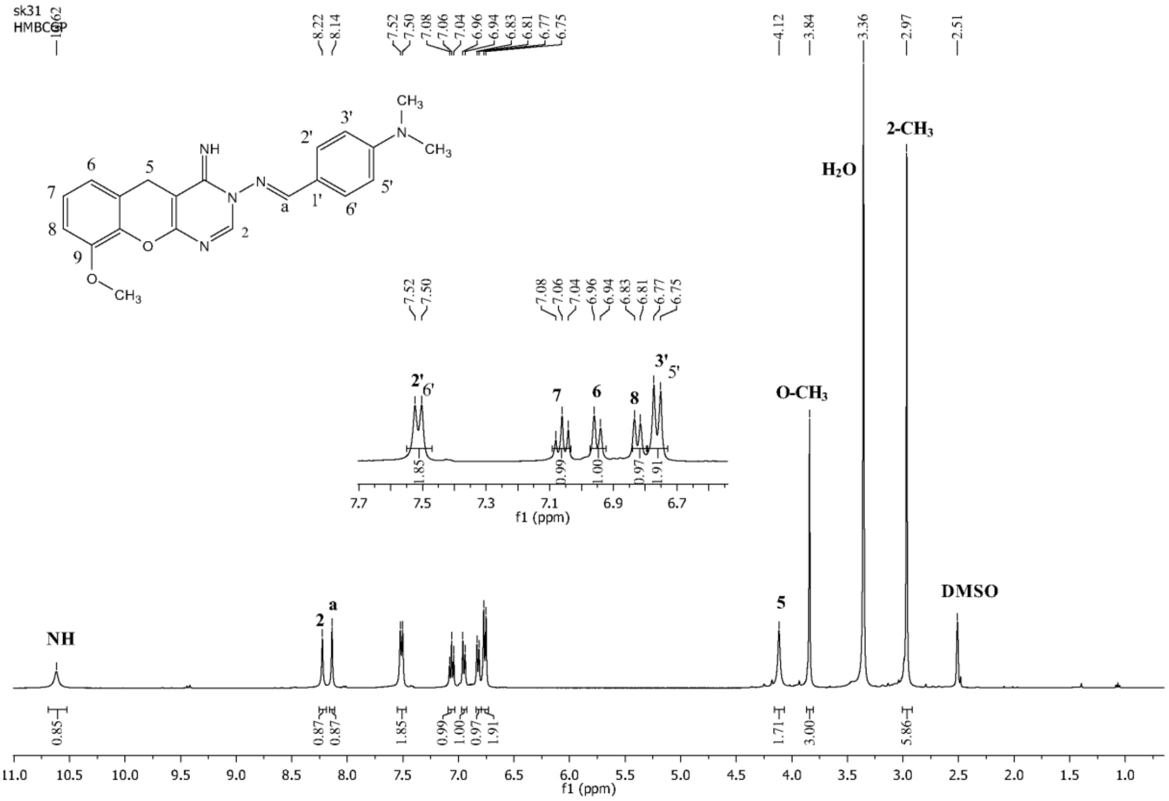

Details on the ^{1}H NMR (300 MHz, DMSO-d_6) spectrum of compound (**10g**).

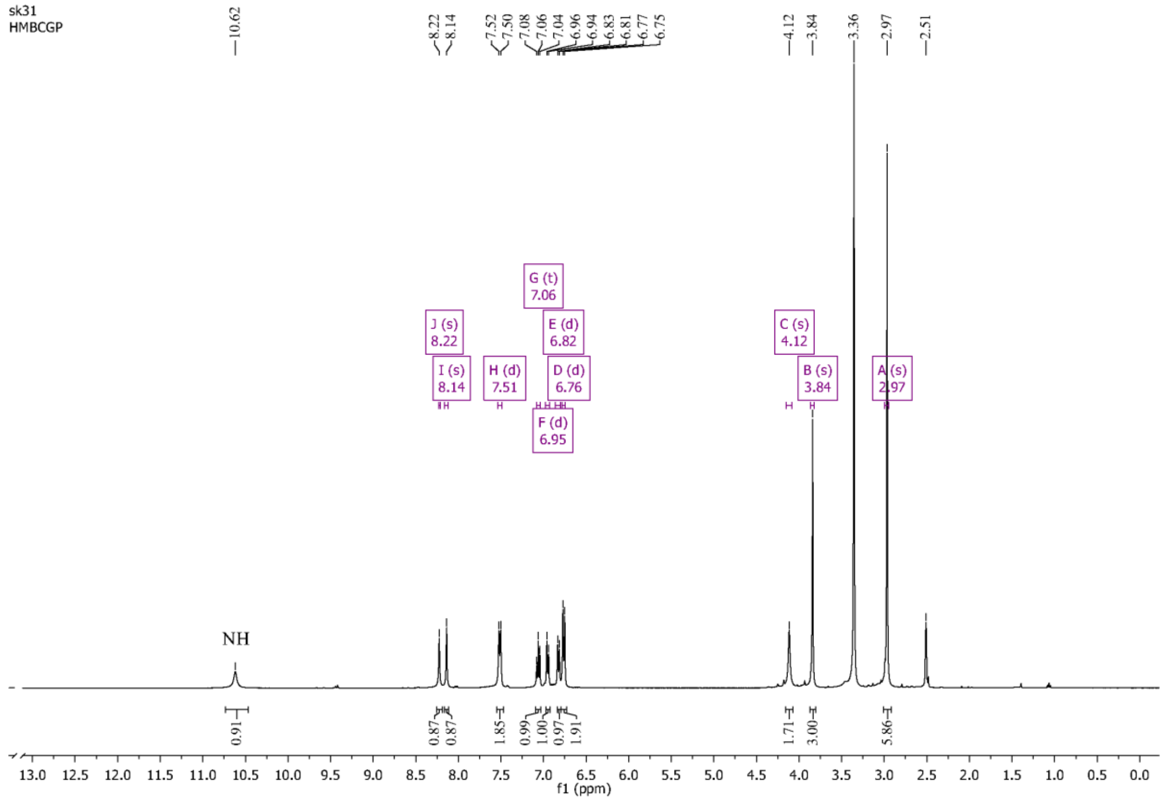

^{13}C NMR (75 MHz, DMSO-d_6) spectrum of compound (**10g**).

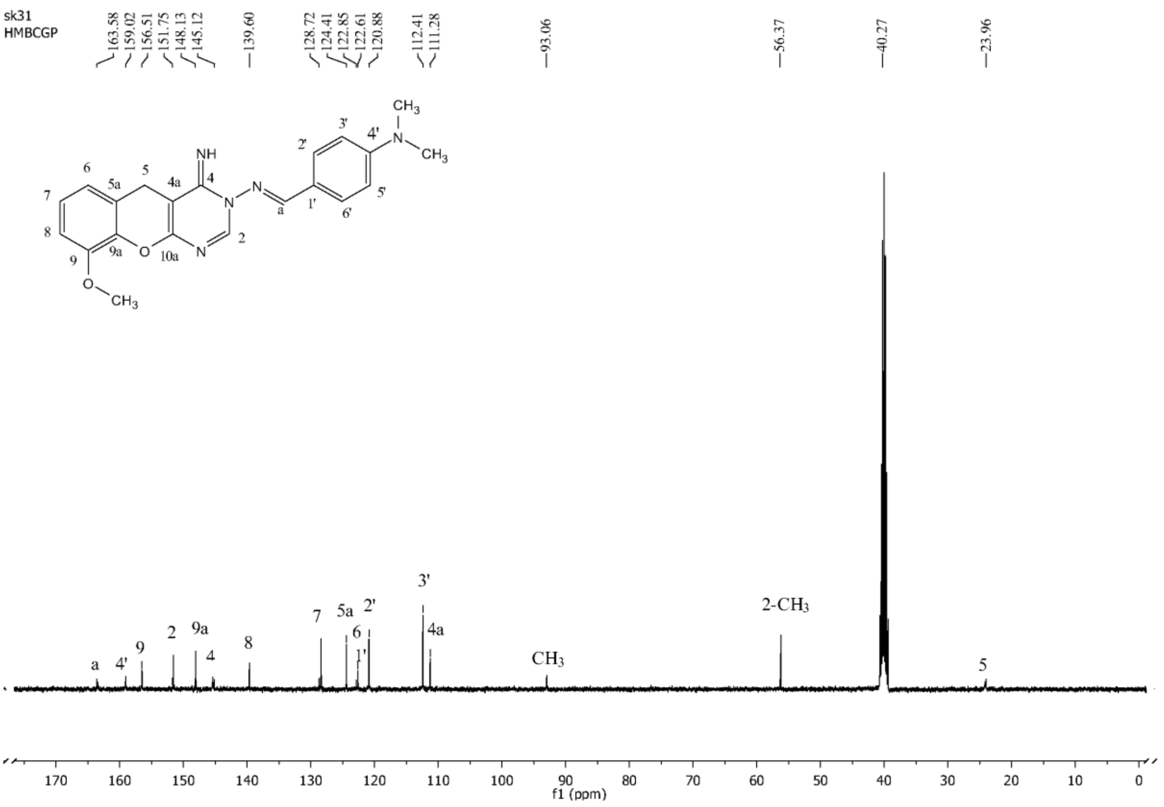

MS spectrum of compound (**10g**).

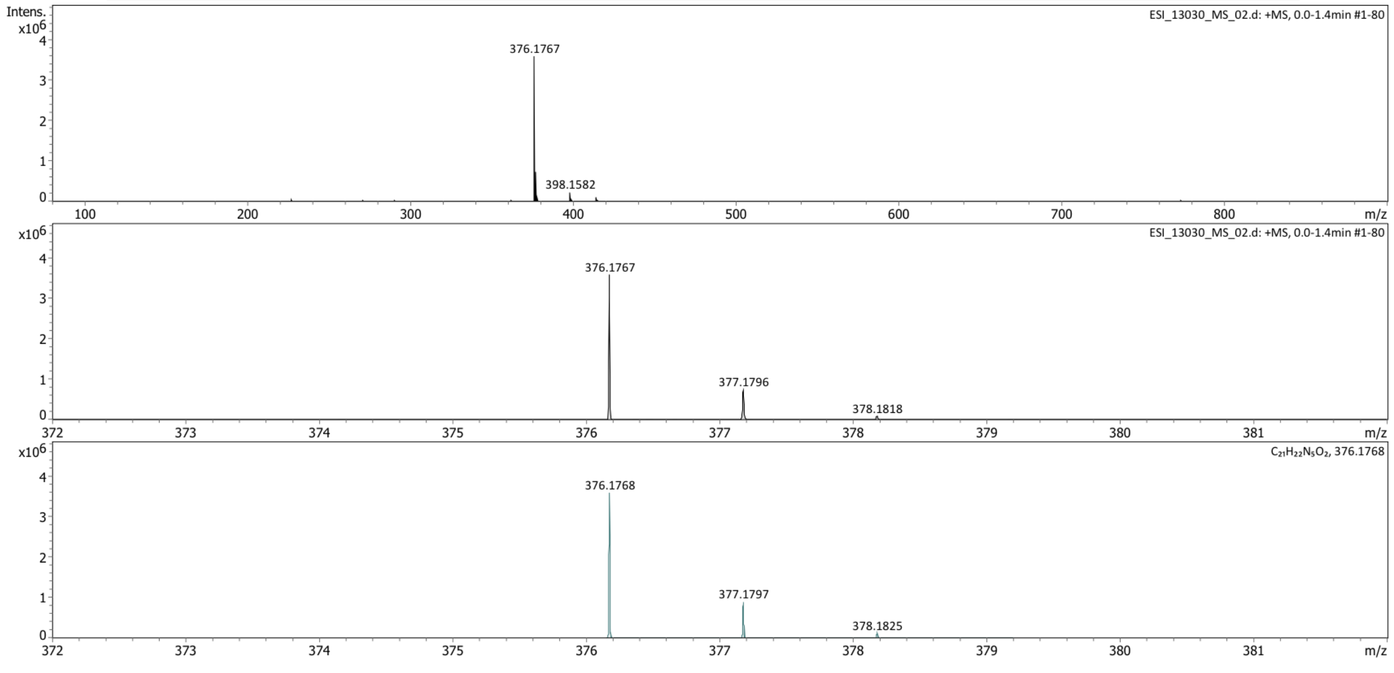

^{1}H NMR (300 MHz, DMSO-d_6) spectrum of compound (**10h**).

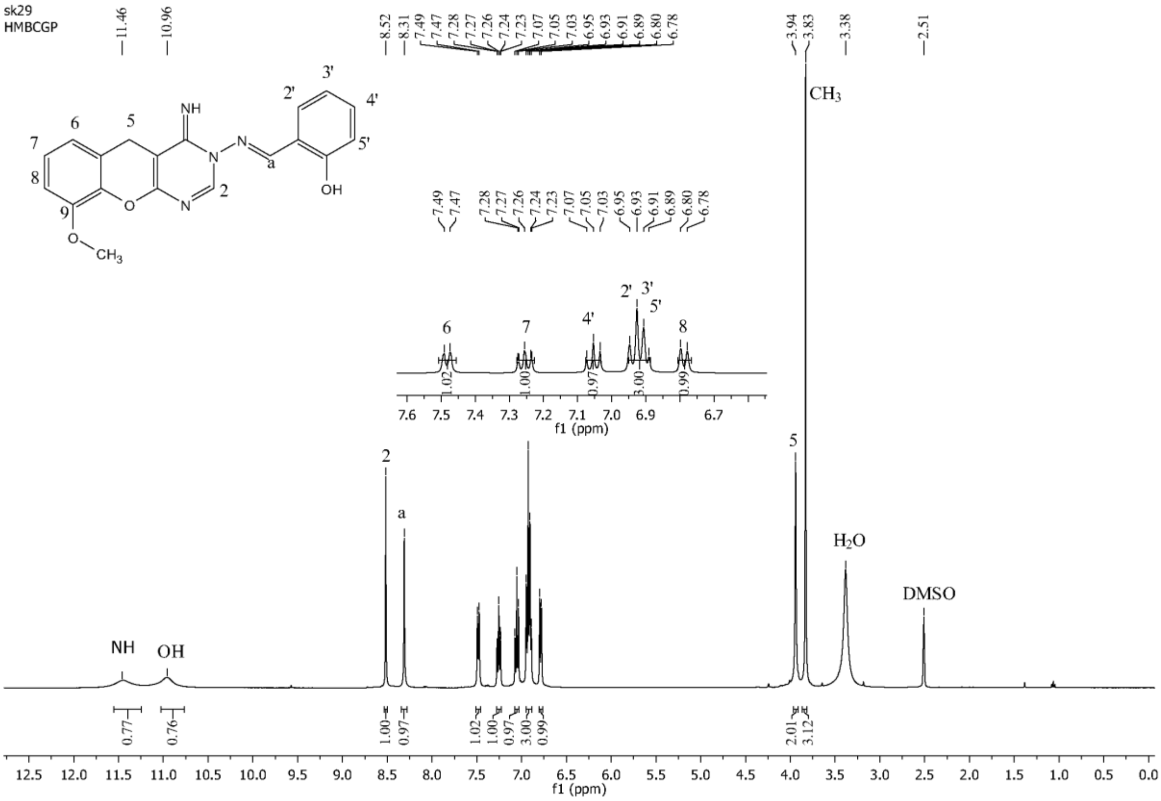

Details on the ^{1}H NMR (300 MHz, DMSO-d_6) spectrum of compound (**10h**).

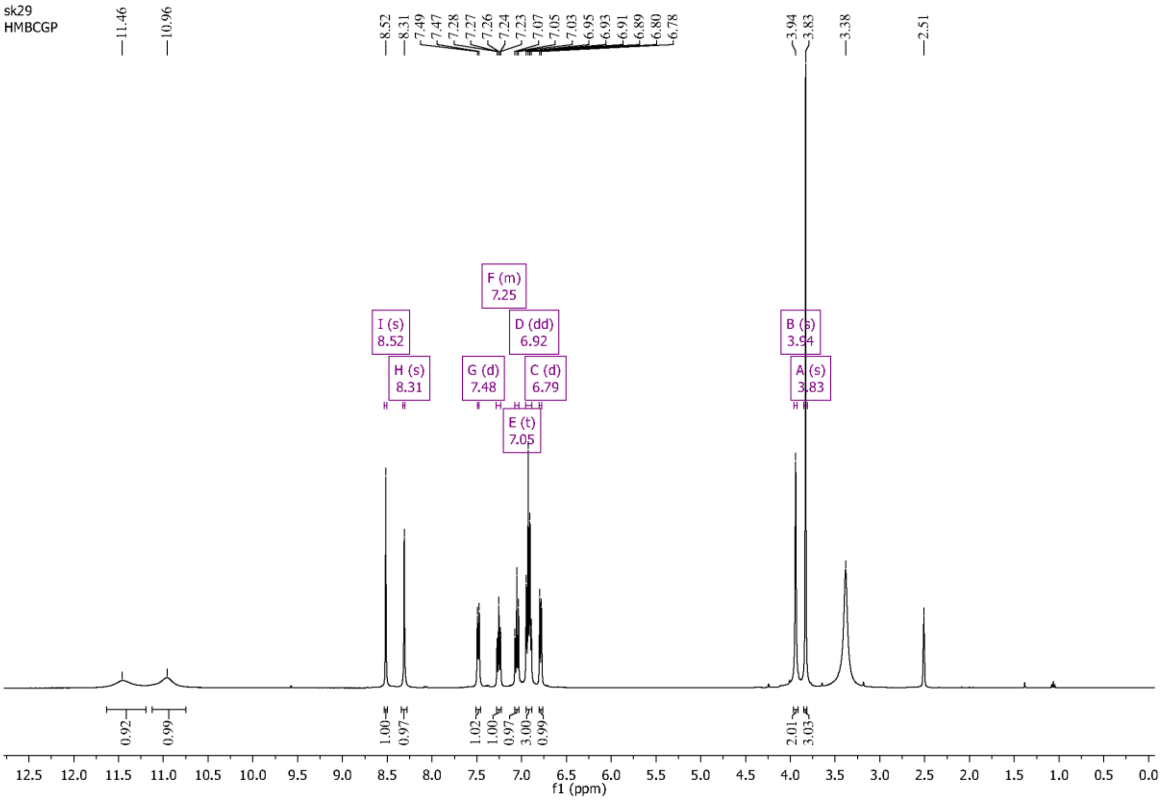

^{13}C NMR (75 MHz, DMSO-d_6) spectrum of compound (**10h**).

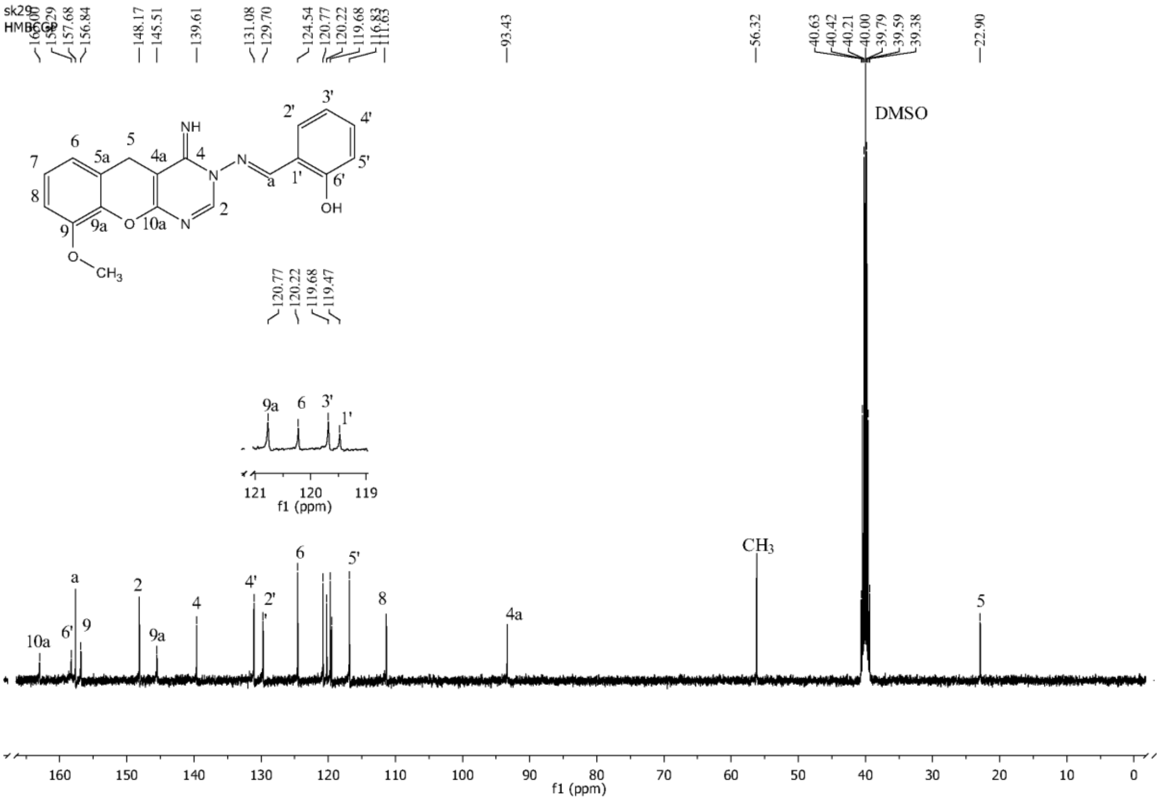

MS spectrum of compound (**10h**).

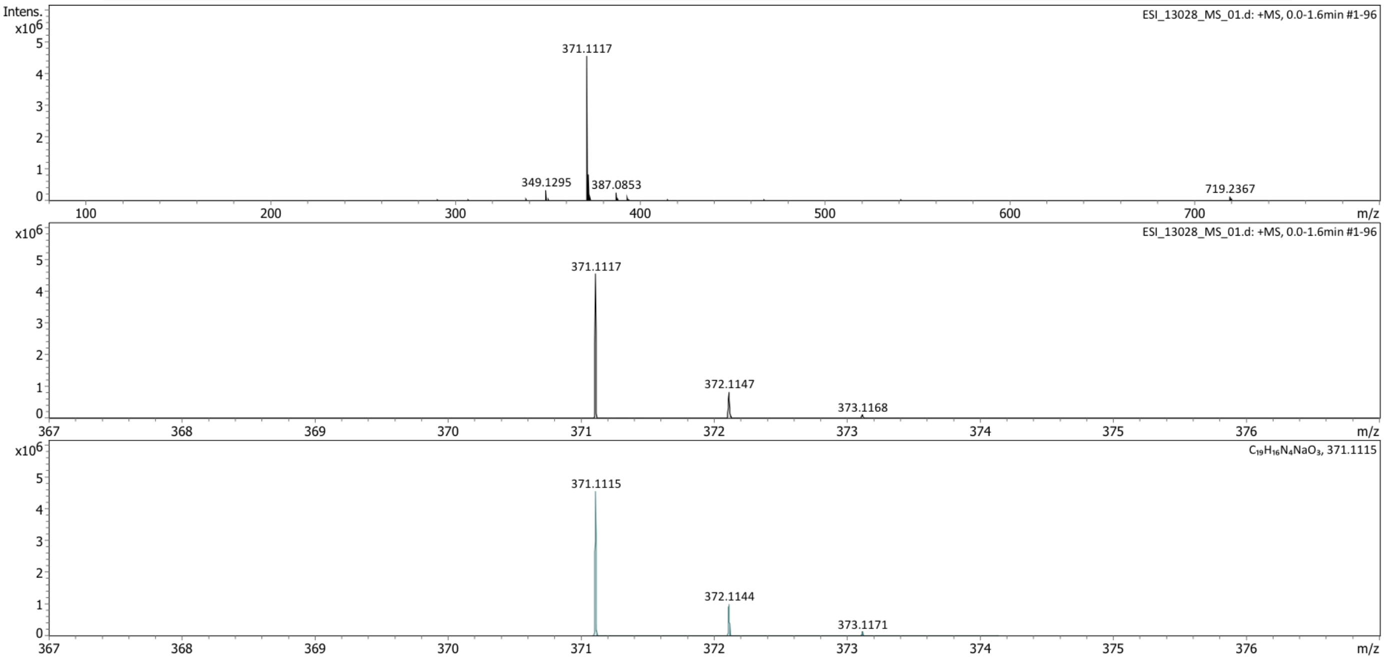

^{1}H NMR (300 MHz, DMSO-d_6) spectrum of compound (**10i**).

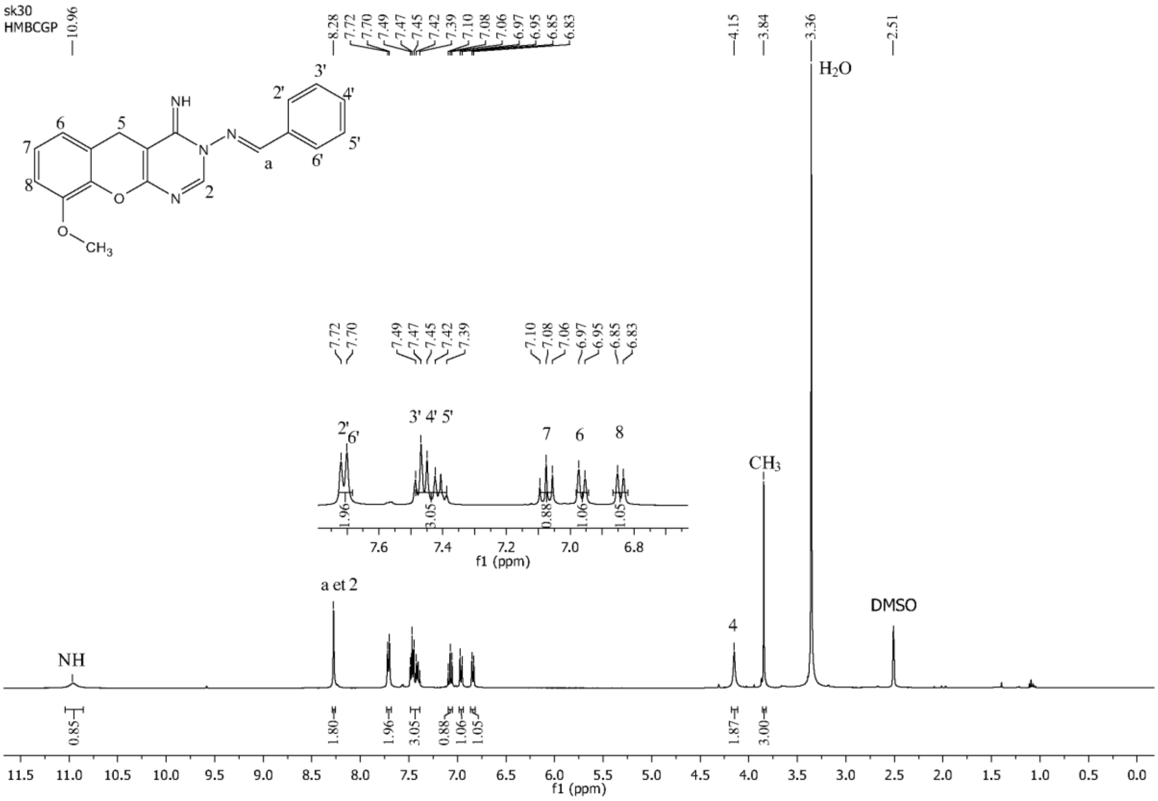

Details on the ^{1}H NMR (300 MHz, DMSO-d_6) spectrum of compound (**10i**).

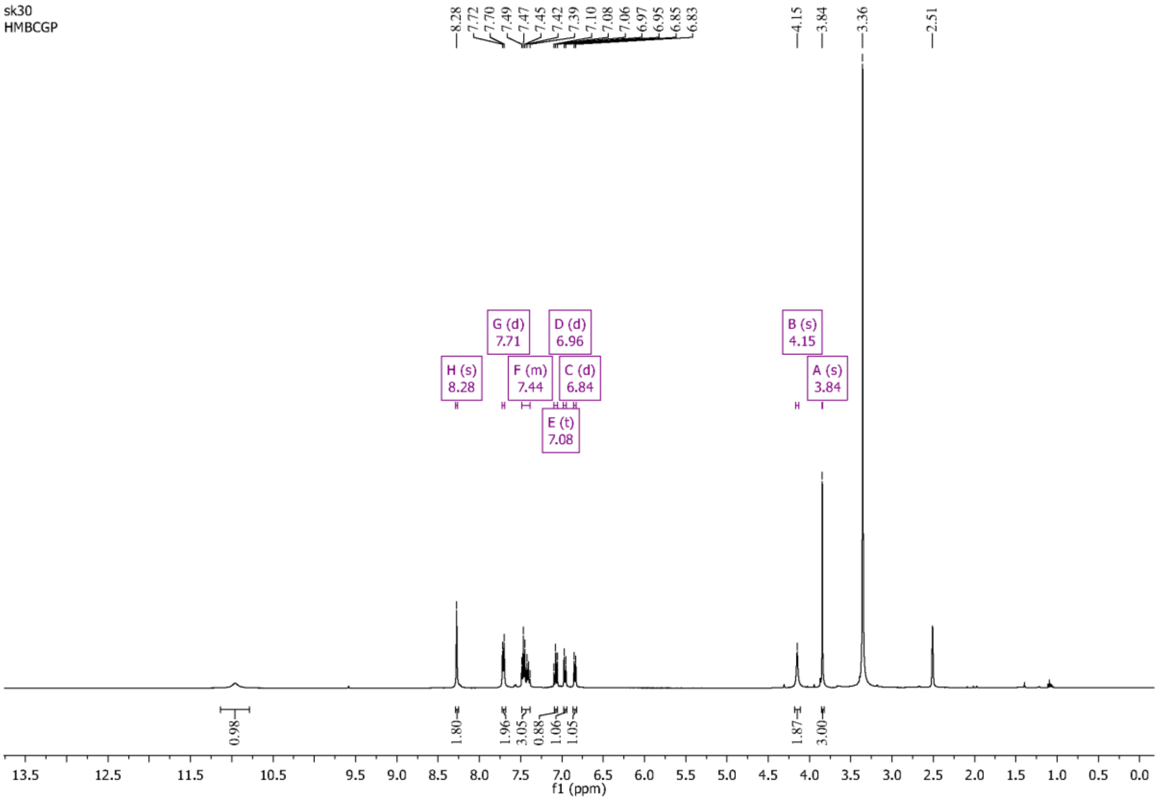

^{13}C NMR (75 MHz, DMSO-d_6) spectrum of compound (**10i**).

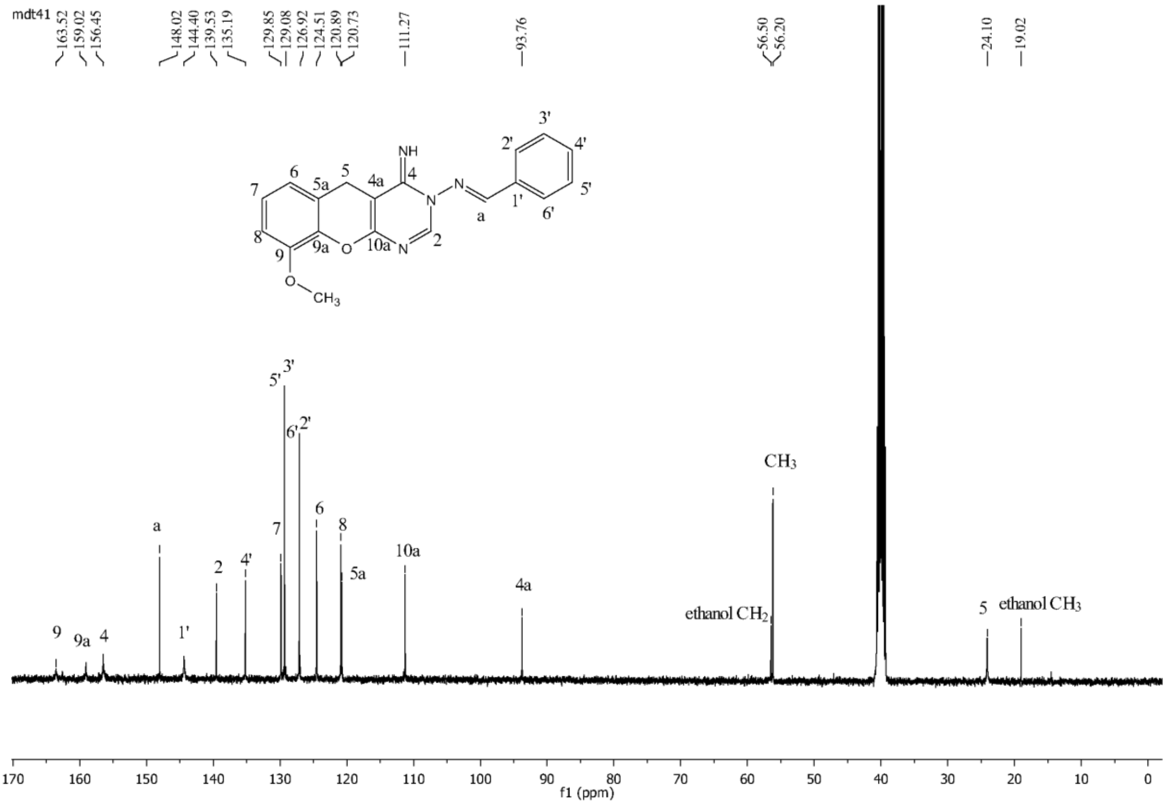

MS spectrum of compound (**10i**).

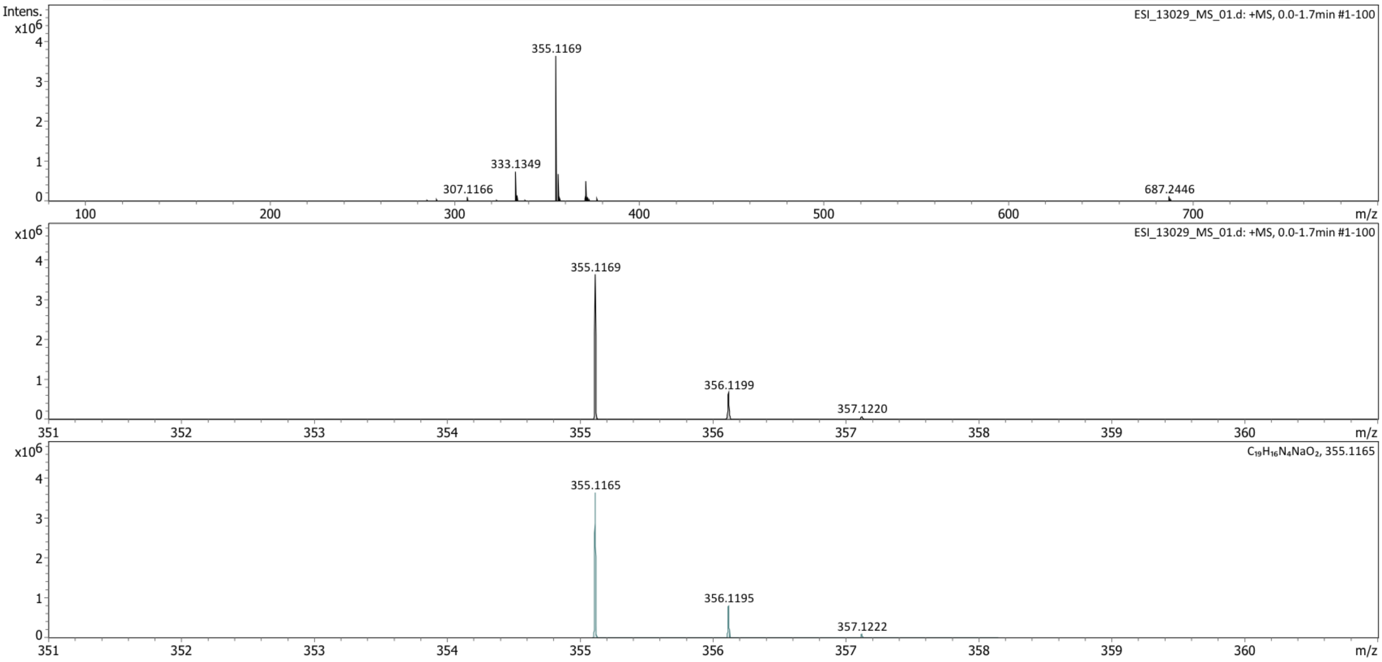

^{1}H NMR (300 MHz, DMSO-d_6) spectrum of compound (**10j**).

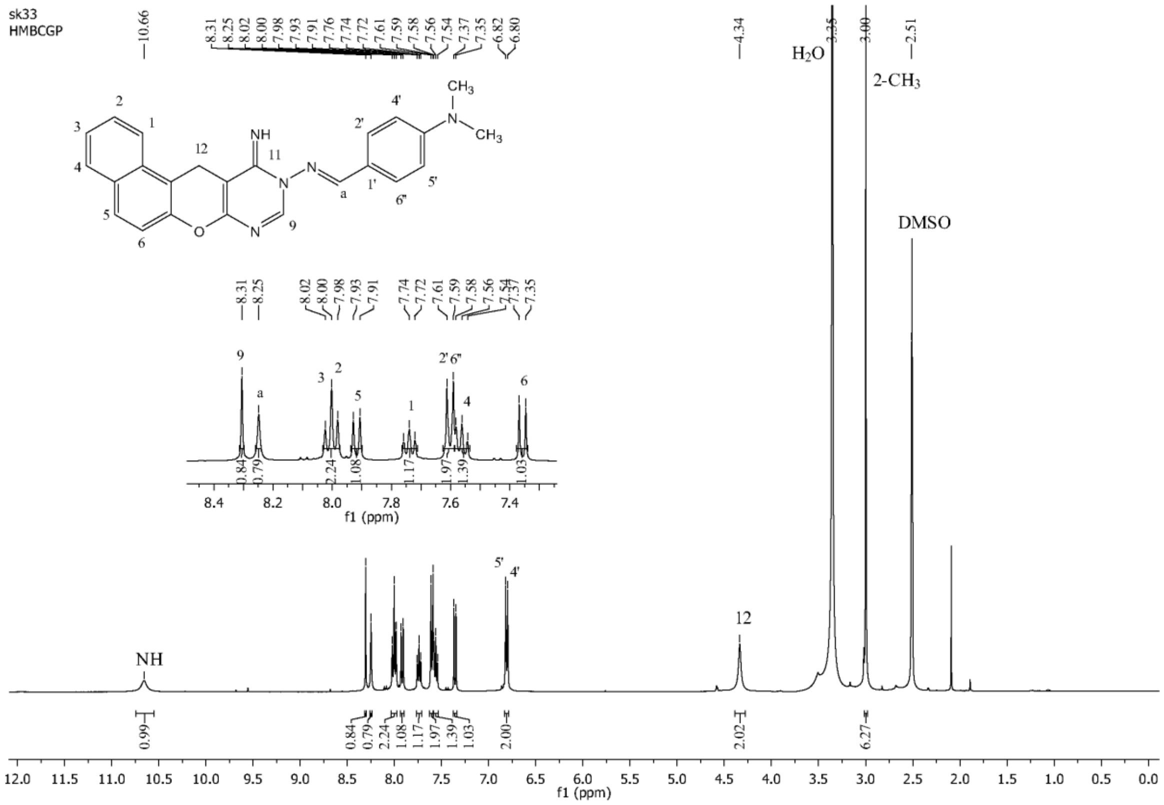

Details on the ^{1}H NMR (300 MHz, DMSO-d_6) spectrum of compound (**10j**).

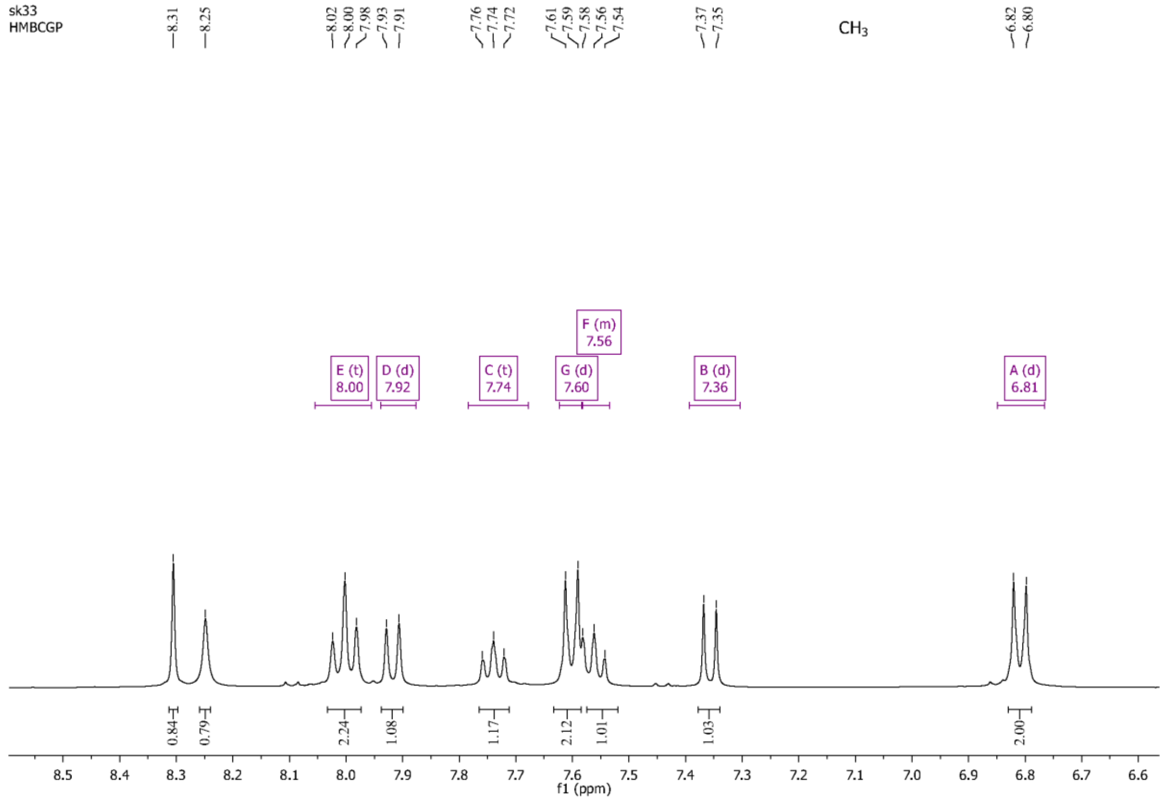

^{13}C NMR (75 MHz, DMSO-d_6) spectrum of compound (**10j**).

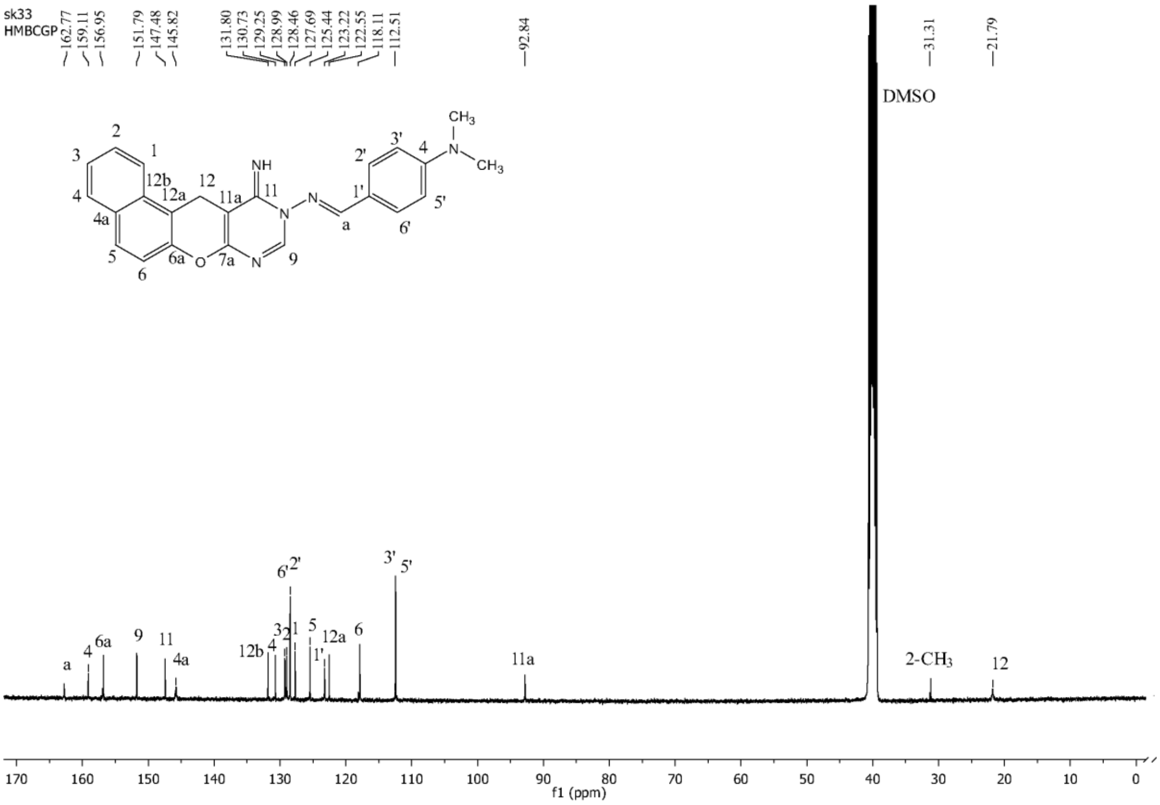

MS spectrum of compound (**10j**).

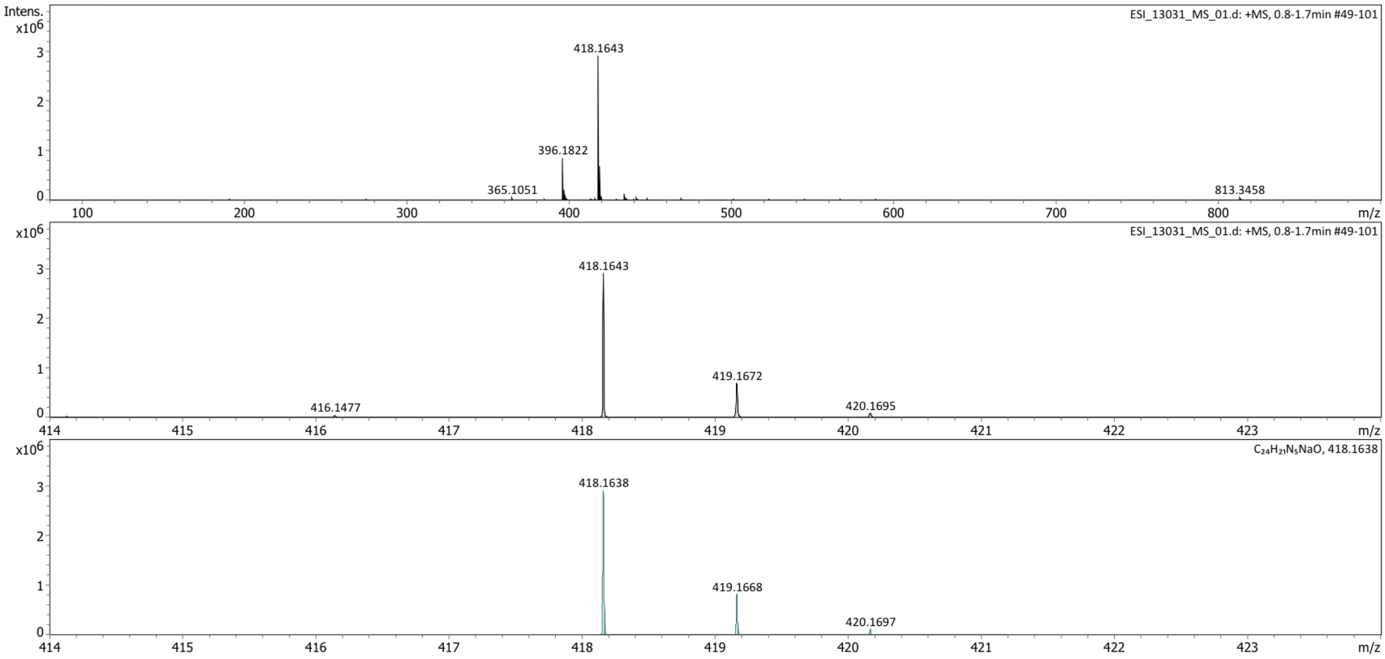

^{1}H NMR (300 MHz, DMSO-d_6) spectrum of compound (**10k**).

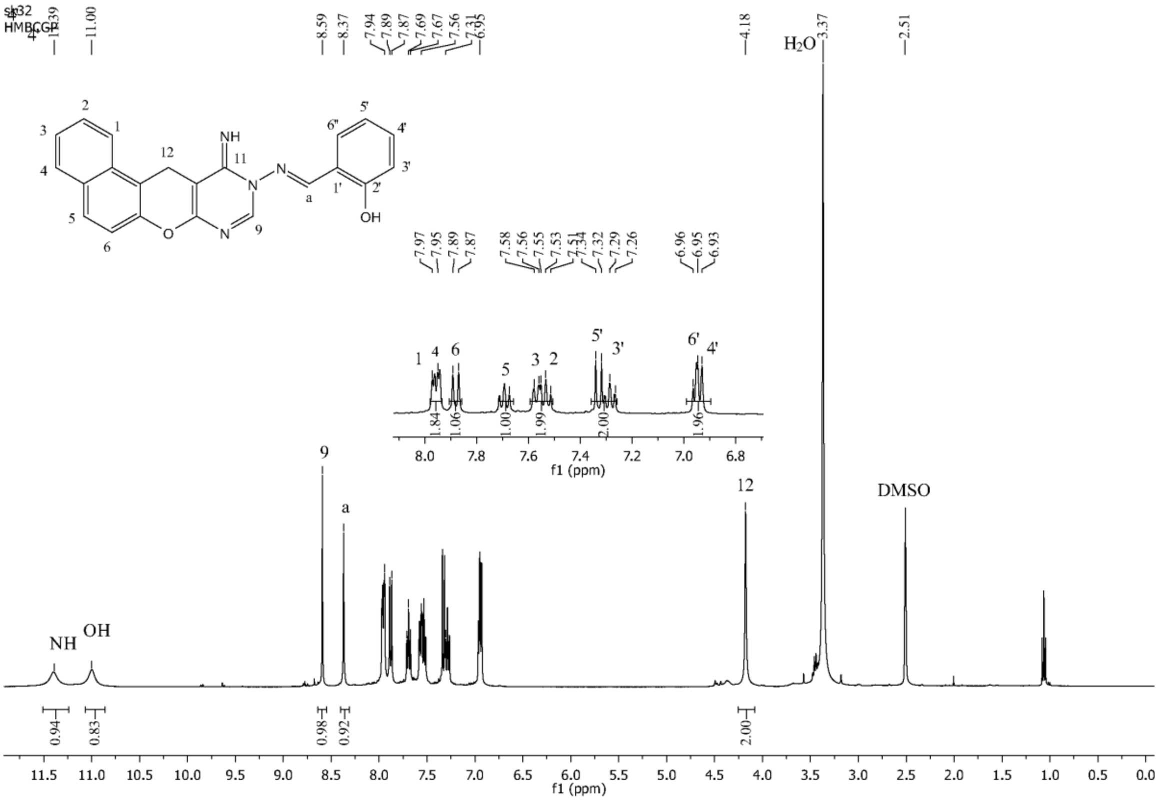

Details on the ^{1}H NMR (300 MHz, DMSO-d_6) spectrum of compound (**10k**).

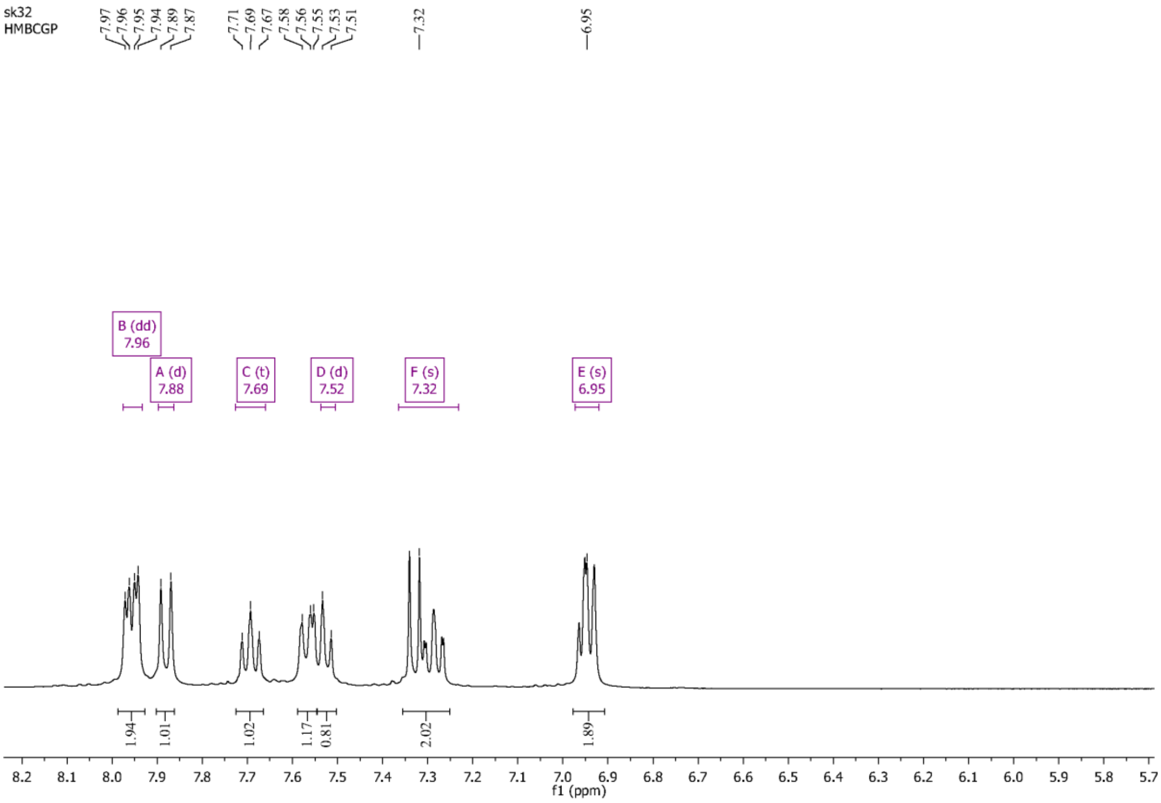

^{13}C NMR (75 MHz, DMSO-d_6) spectrum of compound (**10k**).

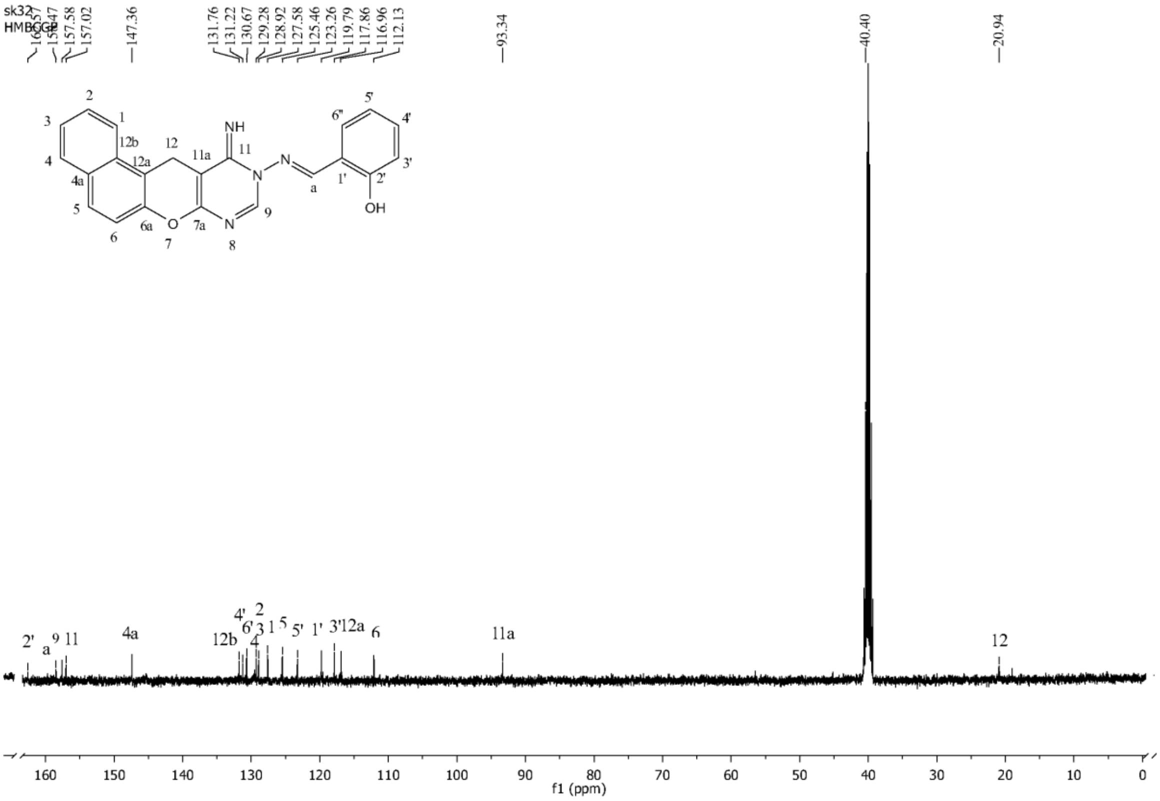

MS spectrum of compound (**10k**).

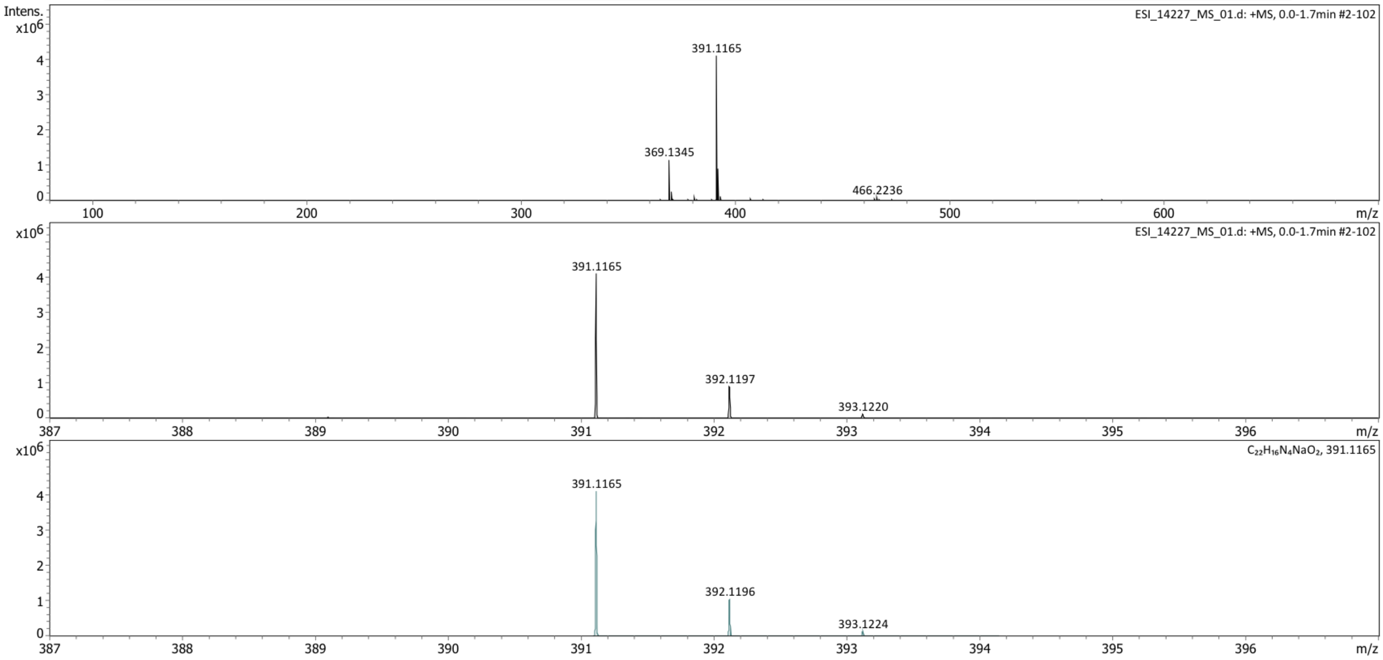

^{1}H NMR (300 MHz, DMSO-d_6) spectrum of compound (**10l**).

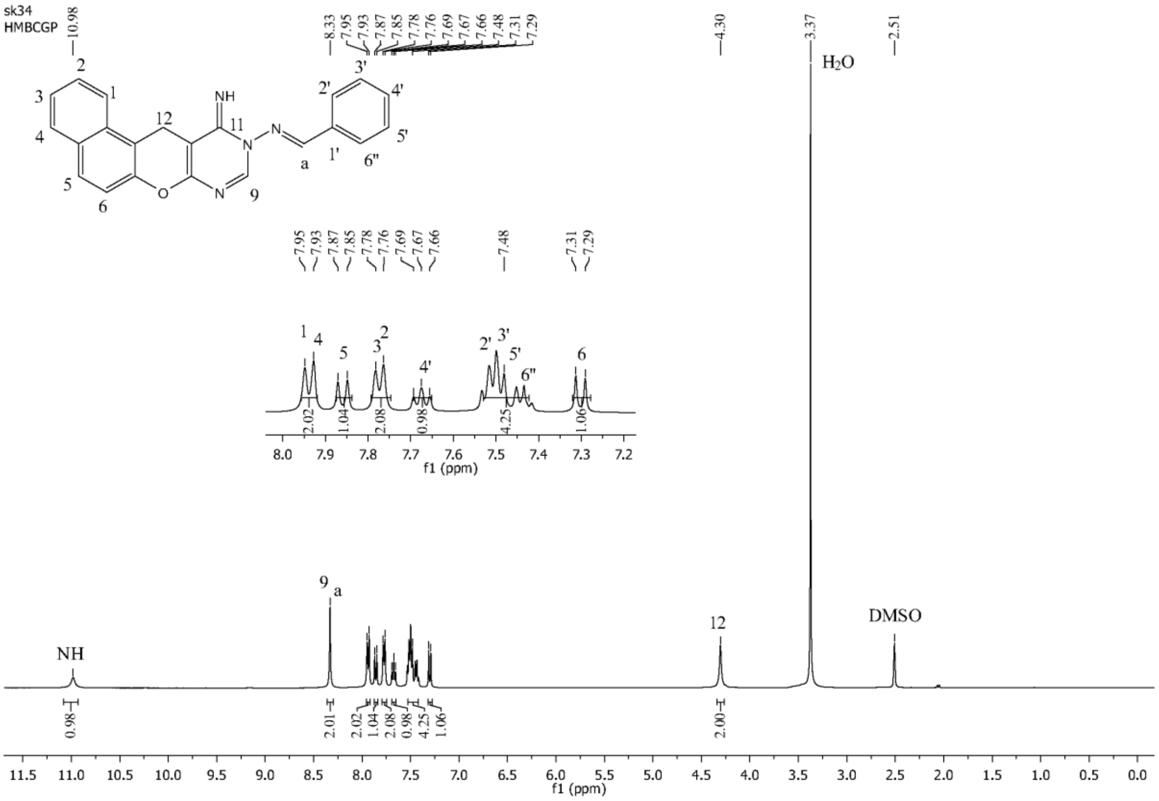

Details on the ^{1}H NMR (300 MHz, DMSO-d_6) spectrum of compound (**10l**).

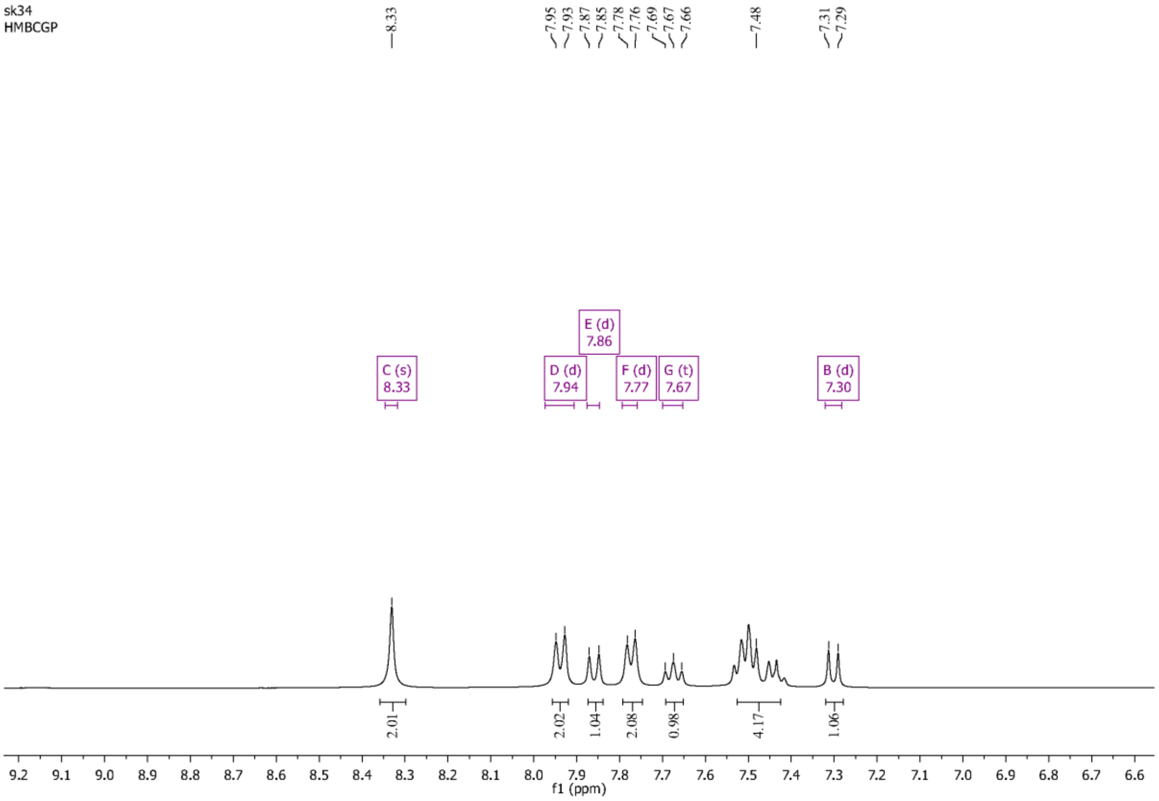

^{13}C NMR (75 MHz, DMSO-*d*$_6$) spectrum of compound (**10l**).

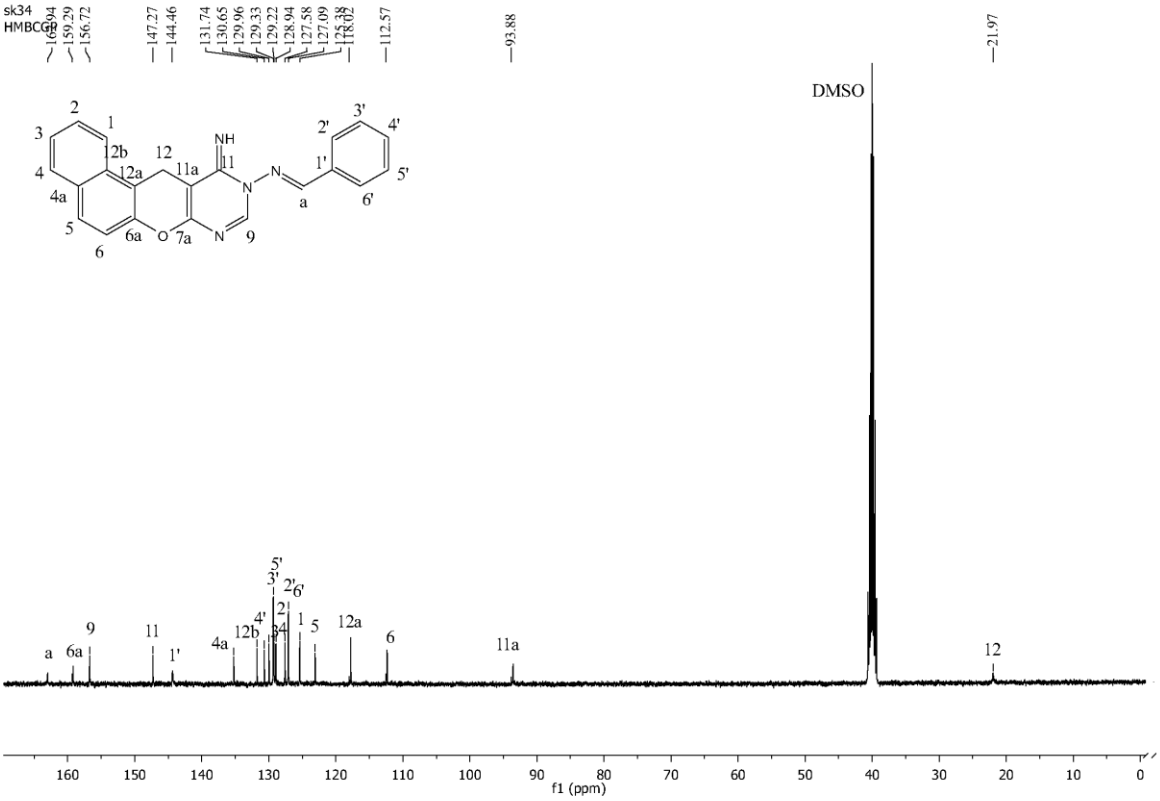

MS spectrum of compound (**10l**).

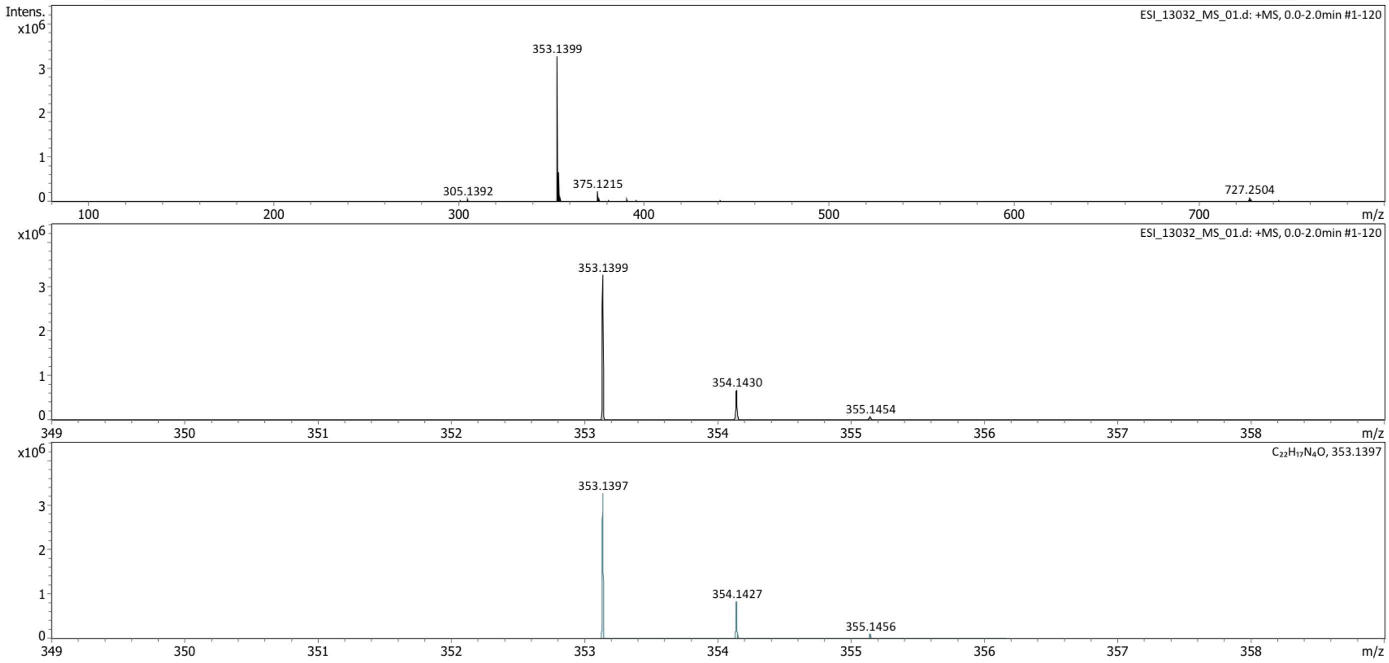

Supplement: Supplementary file 1 [file pharmaceuticals-17-00458-s001.zip › pharmaceuticals-2900496-supplementary.pdf]
